# *Herba Lysimachiae* Polysaccharide‐Modified Selenium Nanoparticles Alleviate Oxidative Injury in Kidney Stones via TOMM22‐Regulated Mitophagy Activation

**DOI:** 10.1002/advs.75784

**Published:** 2026-05-19

**Authors:** Junyi Yang, Guoruiyu Lyu, Wenlong Wan, Dongfeng Yuan, Jiabo Li, Yongqi Wang, Baokang Wang, Zhilong Ma, Yuanyuan Yang, Yang Xun, Xiao Yu

**Affiliations:** ^1^ Department of Urology, Institute of Urology, Tongji Hospital, Tongji Medical College Huazhong University of Science and Technology Wuhan China

**Keywords:** Herba Lysimachiae polysaccharide, kidney stones, mitophagy, selenium nanoparticles, TOMM22

## Abstract

Pharmacological treatments for kidney stones remain limited, with oxidative stress and injury to renal tubular epithelial cells (RTECs) as key pathological drivers. We prepared a novel *Herba Lysimachiae* polysaccharide‐modified selenium nanoparticle (HLP‐SeNPs) formulation that enhances the stability and antioxidant activity of selenium nanoparticles (SeNPs) to alleviate oxalate‐induced renal injury. In vivo and in vitro studies showed that HLP‐SeNPs possess pronounced anti‐oxidative stress capacity, restore mitochondrial membrane potential, alleviate mitochondrial damage, reduce RTEC death, and inhibit renal calcium oxalate (CaOx) crystal deposition. Mechanistically, HLP‐SeNPs downregulate TOMM22 (translocase of the outer mitochondrial membrane 22, a core TOM complex subunit) to activate PINK1‐Parkin‐mediated mitophagy, thereby effectively limiting oxidative stress‐induced injury at its source. These findings indicate that HLP‐SeNPs exert substantial renoprotective effects and prevent CaOx stone formation, providing an experimental foundation for mitochondria‐targeted nanotherapeutics in kidney stone disease and highlighting the potential of integrating traditional Chinese medicine with nanomaterials.

## Graphical abstract

HLP‐SeNPs, a *Lysimachia christinae* polysaccharide‐modified selenium nanoparticle, treat kidney stones by downregulating TOMM22 expression, which reduces PINK1 translocation into mitochondria and promotes its retention on the outer mitochondrial membrane. This restores PINK1/Parkin‐mediated mitophagy, suppresses oxidative stress in renal tubular epithelial cells, and ultimately repairs oxalate‐induced kidney injury.

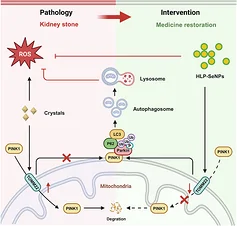

## 1 Introduction

Kidney stone disease (KSD) has emerged as an increasingly serious global public health issue, with its prevalence rising significantly over the past three decades. In particular, the incidence of KSD has nearly tripled in the United States, resulting in a substantial healthcare burden [1]. Beyond acute pain, patients with KSD face significantly elevated risks of developing hypertension, chronic kidney disease, and even end‐stage renal disease [2]. While surgical intervention remains the primary treatment for KSD, effective pharmacological therapies targeting the condition are still lacking. It is widely recognized that oxidative stress plays a crucial role in the pathogenesis of kidney stone formation. Excessive reactive oxygen species (ROS) production leads to damage to renal tubular epithelial cells (RTECs), creating favorable conditions for the adhesion of calcium oxalate (CaOx) crystals, which triggers inflammation and promotes stone formation [3]. In this context, various antioxidants, including vitamin E, tea polyphenols, and sulforaphane, have shown promise in preventing kidney stone formation [4, 5, 6]. However, the clinical application of these traditional antioxidants is constrained by their low bioavailability and poor targeting specificity, emphasizing the need for the development of novel, efficient, and targeted antioxidant therapies for KSD.

While other nanozymes have been shown to combat KSD primarily through direct ROS scavenging and suppression of inflammatory cascades, Selenium nanoparticles (SeNPs) have attracted significant attention due to their unique biological properties, including excellent biocompatibility, relatively low toxicity, and superior antioxidant capacity [7, 8, 9]. Studies have demonstrated that SeNPs effectively alleviate oxidative stress and enhance the activity of antioxidant enzymes such as glutathione peroxidase [10]. Moreover, chloroquine‐loaded SeNPs mitigate nephrolithiasis by inhibiting NF‐κB phosphorylation, suppressing NLRP3 inflammasome activation, and reducing crystal deposition and renal injury [11]. Nevertheless, the intrinsic instability of SeNPs presents a major obstacle to their clinical translation, and their long‐term toxicity and detailed metabolic mechanisms in vivo remain poorly understood. Recent advancements in polysaccharide modification strategies have significantly improved the performance of SeNPs. Polysaccharide coatings not only enhance the physical stability, biological activity, and biocompatibility of SeNPs but also provide them with exceptional anti‐aggregation capabilities and increased cellular uptake efficiency, while maintaining and synergizing their inherent antioxidant and anti‐inflammatory functionalities [12, 13, 14]. For example, selenium‐modified astragalus polysaccharides and selenium‐modified corn silk polysaccharides have been successfully synthesized, and their therapeutic potential in KSD has been explored [15, 16].

*Lysimachia christinae*, a traditional Chinese medicine listed in the “Pharmacopoeia of the People's Republic of China,” has a long history of use for its diuretic and detoxification properties, owing to its natural origin, low toxicity, and minimal drug residue [17]. Modern pharmacological studies have confirmed that *Lysimachia christinae* possesses a variety of bioactivities, including anti‐stone, anti‐inflammatory, and antioxidant effects [18, 19, 20]. *Herba Lysimachiae* polysaccharide (HLP), extracted from *Lysimachia christinae*, are among its most active components and have demonstrated significant therapeutic potential in regulating abnormal cholesterol metabolism, improving cholesterol gallstones and hyperlipidemia, and exhibiting antiviral activity, among other benefits [21, 22]. Leveraging advantages of HLP, this study innovatively designed and synthesized HLP‐modified selenium nanoparticles (HLP‐SeNPs). We anticipated that modifying SeNPs with HLP would not only substantially enhance their biocompatibility and stability but also achieve synergistic improvements in their antioxidant activity, providing a novel therapeutic strategy for KSD treatment.

The kidney, being one of the organs with the highest mitochondrial density in the human body, has a high energy demand and continuous oxidative metabolism, making it particularly vulnerable to ROS imbalance and mitochondrial injury [23, 24]. Mitophagy, as a key mechanism for maintaining mitochondrial quality in RTECs, selectively eliminates dysfunctional mitochondria to counteract excess ROS generation, lipid peroxidation, and mitochondrial dysfunction associated with hyperoxaluria. This process effectively suppresses CaOx crystal deposition and reduces the formation of Randall's plaques [25, 26, 27]. The PINK1‐Parkin pathway is considered the central signaling pathway initiating mitophagy, and evidence suggests that it may be implicated in the pathogenesis of KSD [28, 29]. Despite its critical role in KSD pathophysiology, there remains a lack of nanoparticle‐based therapeutic strategies specifically targeting mitophagy for KSD intervention.

Against this backdrop, this study aimed to develop a novel HLP‐SeNP formulation and systematically investigate its therapeutic effects and molecular mechanisms in KSD. We first synthesized and comprehensively characterized the physicochemical properties and biological activities of HLP‐SeNPs. Subsequently, using an in vitro cell model and an in vivo glyoxylate‐induced KSD model, we systematically evaluated the protective effects of HLP‐SeNPs against renal injury and oxidative stress. On this basis, this study introduced a novel mechanism whereby HLP‐SeNPs alleviate oxalate‐induced ROS generation and RTEC injury by regulating the expression of the key molecule translocase of the outer mitochondrial membrane 22 (TOMM22) to activate the PINK1‐Parkin‐mediated mitophagy pathway. These findings provide novel therapeutic targets and nanoparticle‐based strategies for the clinical treatment of KSD.

## 2 Results

### 2.1 Preparation and Characterization of HLP‐SeNPs

**FIGURE 1:**
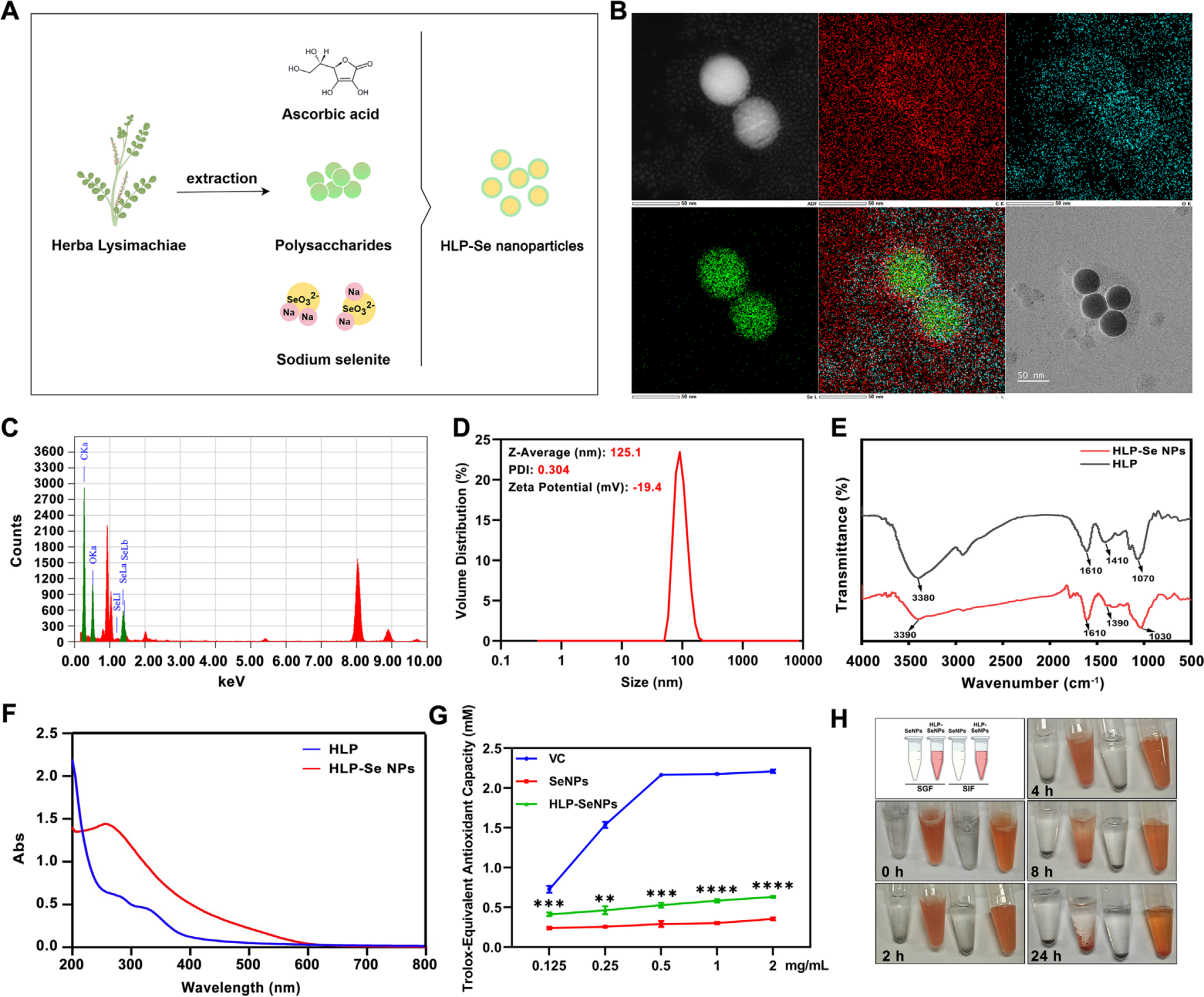
Preparation and characterization of HLP‐SeNPs. (A) Schematic illustration of the preparation of HLP‐SeNPs, with HLP extracted from *Herba Lysimachiae*, sodium selenite as the selenium source and ascorbic acid as the reducing agent. (B) TEM image and corresponding EDX elemental mapping of HLP‐SeNPs (C: red; O: cyan; Se: green), scale bar = 50 nm. (C) Full EDX energy spectrum of HLP‐SeNPs, confirming the presence of elemental selenium. (D) Particle size distribution of HLP‐SeNPs measured by DLS, with the average size, PDI and zeta potential labeled. (E) FTIR spectra of native HLP and synthesized HLP‐SeNPs. (F) UV‐Vis absorption spectra of HLP and HLP‐SeNPs. (G) Trolox‐equivalent T‐AOC comparison between SeNPs and HLP‐SeNPs; ^**^ *p* < 0.01, ^***^ *p* < 0.001, ^****^ *p* < 0.0001. Vitamin C (VC) was used as a positive control. (H) Colloidal stability of SeNPs and HLP‐SeNPs in SGF and SIF after incubation for 0, 2, 4, 8 and 24 h.

The synthesis of HLP‐SeNPs is depicted in Figure 1A. Transmission electron microscopy (TEM) revealed that the as‐prepared HLP‐SeNPs exhibited uniform monodisperse spherical morphology, with a dry‐state particle diameter of approximately 40 nm (Figure 1B). Energy‐dispersive X‐ray spectroscopy (EDX) elemental mapping further confirmed that carbon (C), oxygen (O), and selenium (Se) were homogeneously distributed throughout the entire nanoparticles (Figure 1B). A characteristic Se energy peak was clearly detected at 1.379 keV in the full EDX spectrum, with selenium content determined as 15.78 wt.% (Figure 1C), verifying the successful fabrication of selenium‐containing nanoparticles. Dynamic light scattering (DLS) analysis showed that HLP‐SeNPs had a mean hydrodynamic diameter of 125.1 nm, a polydispersity index (PDI) of 0.304, and a negative surface zeta potential of ‐19.4 mV (Figure 1D). Fourier transform infrared (FTIR) confirmed that the HLP‐SeNPs retained key absorption peaks of HLP, with a notable blue shift of the ‐OH absorption peak to 3390 cm^−1^ and a red shift of the C─O─H peak to 1030 cm^−1^, suggesting possible hydrogen bond dissociation and formation of C─O─Se bonds between HLP and SeNPs (Figure 1E). UV‐Vis absorption spectroscopy showed that HLP‐SeNPs had a characteristic absorption peak centered at 260 nm, which was absent in pure HLP, consistent with the formation of elemental selenium nanoparticles (Figure 1F).

The total antioxidant capacity (T‐AOC) assay was performed to compare the antioxidant activity of HLP‐SeNPs and unmodified SeNPs. HLP‐SeNPs exhibited significantly higher antioxidant capacity than SeNPs at all tested concentrations, demonstrating that HLP modification greatly enhanced the antioxidant activity of SeNPs (Figure 1G). Given that HLP‐SeNPs are designed for oral administration against kidney stones, we further evaluated their colloidal stability in physiological gastrointestinal environments. As shown in Figure 1H, HLP‐SeNPs remained stable for 4 and 8 h in simulated gastric fluid (SGF) and simulated intestinal fluid (SIF), respectively, whereas SeNPs aggregated after only 2 h in both conditions. It proved that HLP coating dramatically improved the gastrointestinal stability of SeNPs. These results demonstrate that HLP modification significantly improves both the antioxidant activity and gastrointestinal colloidal stability of SeNPs.

### 2.2 Oral Administration of HLP‐SeNPs Alleviate Glyoxylate‐Induced Kidney Injury via Attenuating Oxidative Stress

**FIGURE 2:**
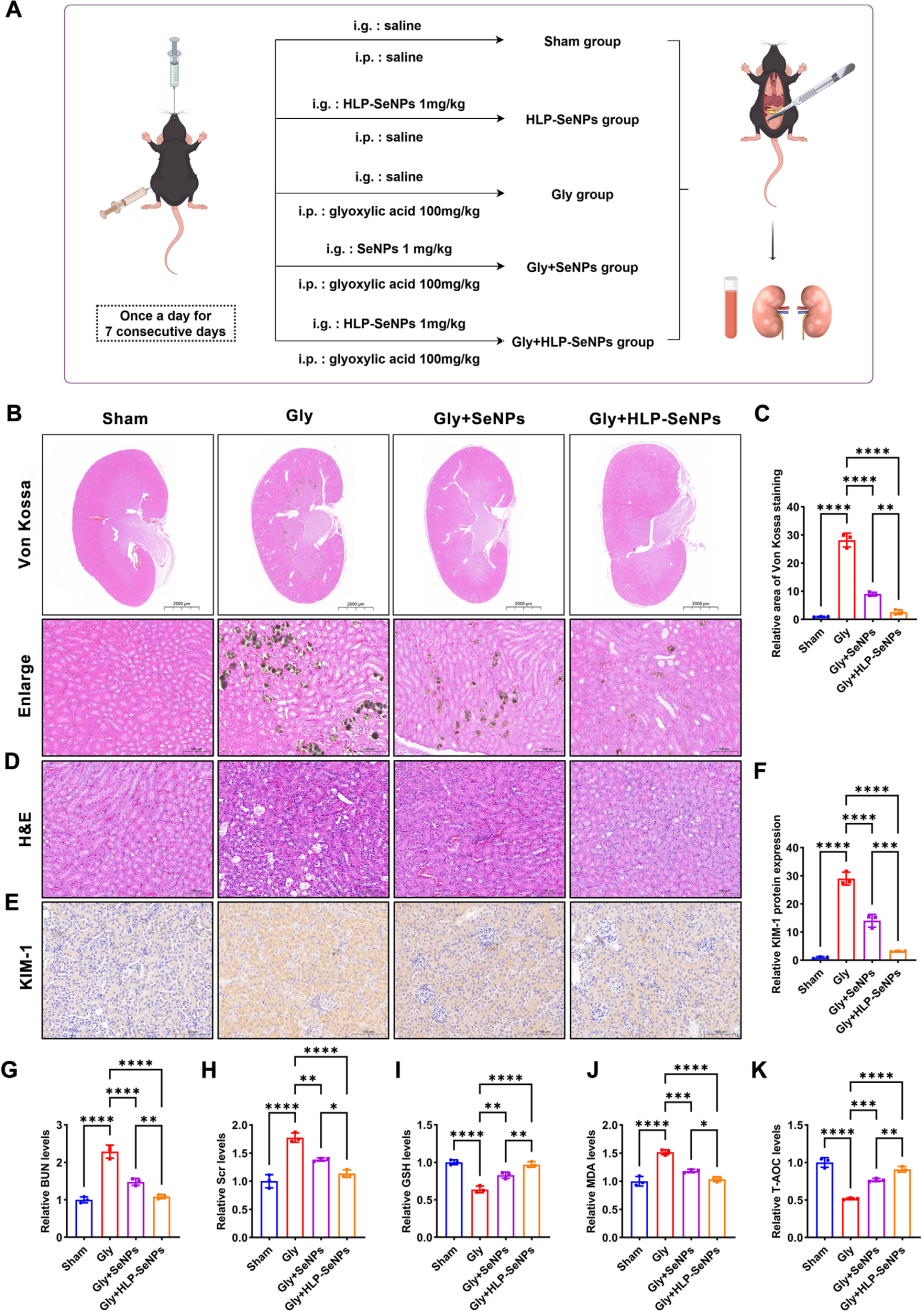
Protective effects of HLP‐SeNPs against glyoxylate‐induced CaOx deposition and oxidative stress in the mouse kidney. (A) Schematic illustration of the in vivo experimental design and animal grouping. (B) Representative Von Kossa staining of kidney tissues from different groups. (C) Semi‐quantitative analysis of the relative Von Kossa positive crystal area. (D) Representative H&E staining of kidney tissues. (E) Representative KIM‐1 immunohistochemical staining of kidney tissues. (F) Semi‐quantitative analysis of relative KIM‐1 protein expression. (G‐K) Relative levels of serum BUN (G), serum Scr (H), renal GSH (I), renal MDA (J), and renal T‐AOC (K) in each group. Data are presented as mean ± SD. ^*^ *p* < 0.05, ^**^ *p* < 0.01, ^***^ *p* < 0.001, ^****^ *p* < 0.0001.

To evaluate the in vivo therapeutic efficacy of HLP‐SeNPs against CaOx kidney injury, we established a glyoxylate‐induced mouse KSD model, with the experimental workflow and animal grouping shown in Figure 2A. Von Kossa staining revealed massive CaOx crystal deposition in the glyoxylate‐treated group. Hematoxylin‐eosin (H&E) staining and immunohistochemistry for kidney injury molecule‐1 (KIM‐1, a sensitive marker of tubular damage) further confirmed that glyoxylate challenge induced severe structural injury and tubular dysfunction. Both SeNPs and HLP‐SeNPs oral administration substantially reduced crystal deposition and alleviated renal impairment, with HLP‐SeNPs displaying significantly greater efficacy than SeNPs (Figure 2B–F). Consistent with histological results, serum biochemical assays showed that HLP‐SeNPs more effectively reduced the levels of renal dysfunction markers blood urea nitrogen (BUN) and serum creatinine (Scr) compared to SeNPs (Figure 2G,h). To characterize the antioxidant effect, we measured oxidative stress markers in kidney tissues. HLP‐SeNPs more efficiently restored glutathione (GSH) level and T‐AOC, while decreasing the lipid peroxidation product malondialdehyde (MDA), confirming the potent antioxidant activity of HLP‐SeNPs (Figures 2I,K). As shown in Figure S1, free HLP treatment did not significantly reduce CaOx crystal deposition, improve renal function, or alleviate oxidative stress relative to the Gly group, confirming that the therapeutic effect is mediated by HLP‐SeNPs rather than free HLP alone.

Additionally, H&E staining and organ function tests confirmed that HLP‐SeNPs exhibited no evident toxicity in major organs including the heart, liver, spleen, lungs, and kidneys (Figure S2). We further investigated the in vivo biodistribution and renal targeting property of HLP‐SeNPs after oral administration, using Cy5‐labeled nanoparticles for fluorescence tracking. Immunofluorescence co‐localization demonstrated that HLP‐SeNPs accumulated in aquaporin 1 (AQP1)‐positive proximal renal tubular cells, the primary site of CaOx crystal deposition (Figure S3A). Sequential in vivo and ex vivo fluorescence imaging, combined with semi‐quantitative analysis of renal fluorescence intensity, showed that renal accumulation of HLP‐SeNPs peaked at 2 h post‐administration and was almost completely cleared within 8 h, while all nanoparticle signal was cleared from other organs within 24 h, confirming rapid clearance with no long‐term retention (Figure S3B).

Collectively, these results demonstrate that HLP‐SeNPs specifically target the kidney after oral administration, exert potent therapeutic effects against glyoxylate‐induced kidney injury by reducing oxidative stress, and exhibit excellent biosafety without systemic toxicity.

### 2.3 Mechanistic Investigation of HLP‐SeNPs Alleviating Oxalate‐Induced Renal Tubular Injury via Regulating Mitophagy

**FIGURE 3:**
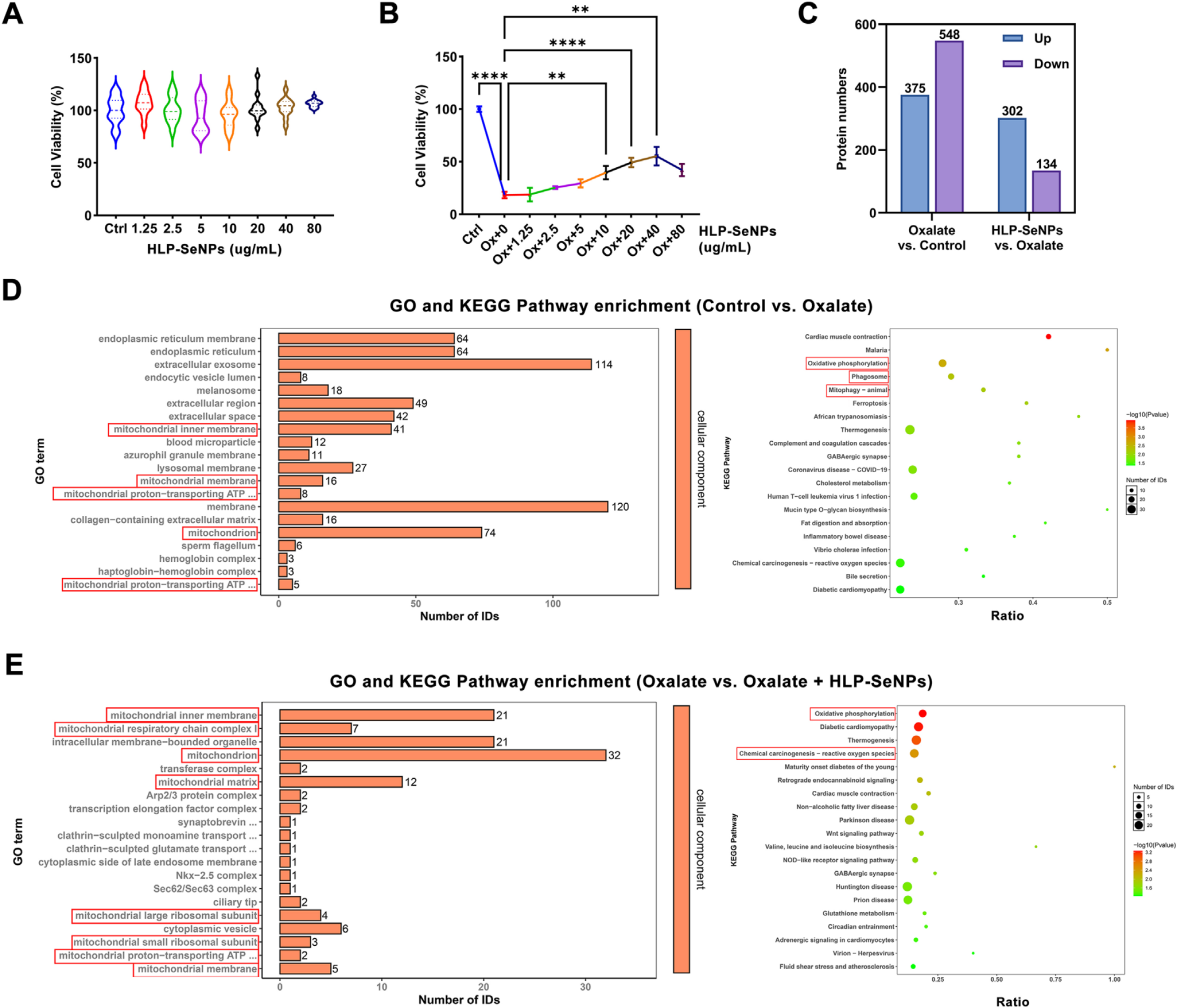
Quantitative proteomics identifies mitophagy as a potential functional pathway. (A) Cell viability of HK‐2 cells treated with gradient concentrations of HLP‐SeNPs alone detected by CCK‐8 assay. (B) Cell viability of HK‐2 cells co‐treated with oxalate and gradient concentrations of HLP‐SeNPs. Data are presented as mean ± SD. ^**^ *p* < 0.01, ^****^ *p* < 0.0001. (C) Bar plot showing the number of upregulated (Up) and downregulated (Down) DEPs in Oxalate vs. Control and HLP‐SeNPs + Oxalate vs. Oxalate comparisons. (D) GO cellular component enrichment (left) and KEGG pathway enrichment (right) of DEPs between Control and Oxalate groups. (E) GO cellular component enrichment (left) and KEGG pathway enrichment (right) of DEPs between Oxalate and Oxalate + HLP‐SeNPs groups. Mitochondrial‐related terms are highlighted with red boxes.

To explore how HLP‐SeNPs protect against oxalate‐induced renal tubular injury, we first established an in vitro oxalate injury model using human proximal tubular epithelial (HK‐2) cells. The results of Cell Counting Kit‐8 (CCK‐8) assays confirmed that treatment with various concentrations of HLP‐SeNPs alone did not significantly affect HK‐2 cell viability (Figure 3A). When co‐treated with sodium oxalate (Ox), HLP‐SeNPs dose‐dependently rescued oxalate‐induced cell death, with significant cytoprotection observed at 10, 20, and 40 µg/mL. We selected 20 µg/mL as the optimal intervention concentration for subsequent experiments based on these results (Figure 3B).

To identify potential regulatory pathways and target proteins of HLP‐SeNPs, we performed Astral‐data‐independent acquisition (Astral‐DIA) quantitative proteomics to compare proteomic profiles across the control, oxalate, and oxalate + HLP‐SeNPs groups. Compared with the control group, 923 differentially expressed proteins (DEPs) were identified in the oxalate group (375 upregulated, 548 downregulated). Compared with the oxalate‐alone group, 436 DEPs were detected in the HLP‐SeNPs co‐treatment group (302 upregulated, 134 downregulated) (Figure 3C). Gene Ontology (GO) enrichment analysis of cellular components showed that DEPs from both comparisons were predominantly enriched in mitochondrial‐related compartments, including the mitochondrial membrane and matrix (Figure 3D,E). Kyoto Encyclopedia of Genes and Genomes (KEGG) pathway enrichment further showed that oxalate exposure mainly affected oxidative phosphorylation, phagosome, and mitophagy pathways, while HLP‐SeNPs intervention also dominantly regulated oxidative phosphorylation and ROS‐related pathways (Figure 3D,E). These bioinformatics results strongly suggested that mitophagy, the core pathway for mitochondrial quality control, may be a key mediator of the renoprotective effect of HLP‐SeNPs.

To test this hypothesis and screen core target proteins, we obtained 121 mitophagy‐related gene sets from the GSEA database (www.gsea‐msigdb.org), and performed overlapping analysis with our proteomic data. First, we extracted 217 DEPs that were differentially expressed in both comparisons, representing proteins whose oxalate‐induced expression alterations were reversed by HLP‐SeNPs intervention (Figure S4A). Intersection of these 217 reversed DEPs with 121 mitophagy‐related genes yielded 5 overlapping candidate targets: TOMM22, RPS27A, GABARAPL2, TBC1D15, and CSNK2B (Figure S4B).

**FIGURE 4:**
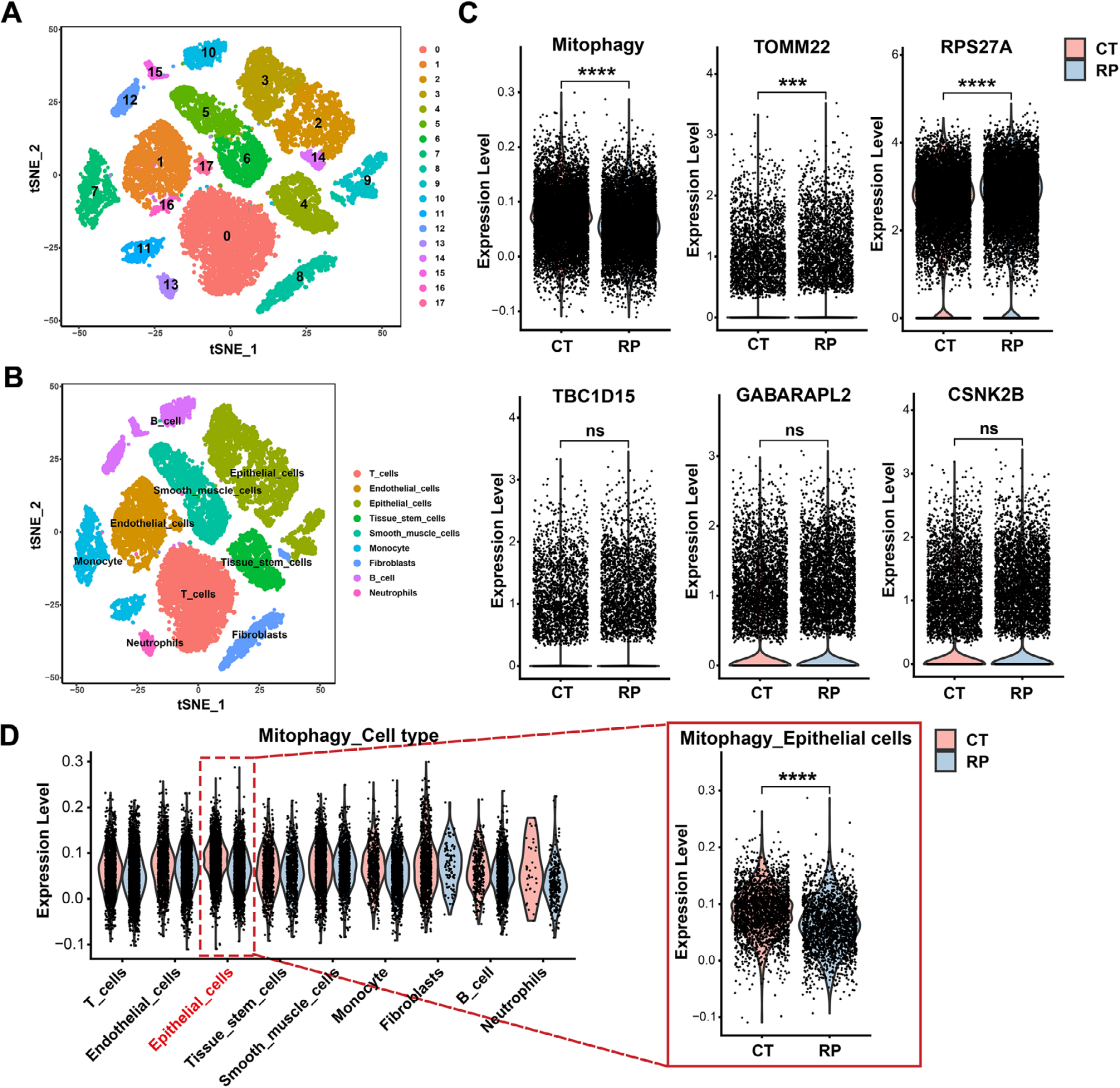
Single‐cell RNA sequencing validation of candidate mitophagy‐related genes in clinical Randall's plaque samples. (A) tSNE plot of all cells from control (CT) and Randall's plaque (RP) tissues. (B) Annotated tSNE plot showing 9 major cell types. (C) Distribution of mitophagy gene set scores and expression levels of five candidate genes between CT and RP groups. (D) Mitophagy gene set scores across all cell types (left), and zoomed comparison of mitophagy score in epithelial cells between CT and RP (right, red box). Data are presented as mean ± SD. ^***^ *p* < 0.0001, ^****^ *p* < 0.0001, ns: no significance.

Randall's plaque (RP) is the well‐recognized precursor lesion of calcium oxalate kidney stones [30]. To validate our candidate genes in clinical samples, we analyzed a public single‐cell RNA sequencing dataset (GSE176155) from human RP lesion and matched normal control kidney tissues. We performed quality control and batch correction, followed by t‐distributed stochastic neighbor embedding (tSNE) for dimensionality reduction and cell clustering (Figure 4A,B). We calculated the mitophagy‐related gene set score for each cell using the AddModuleScore algorithm, and found the overall mitophagy score was significantly lower in RP tissues than in normal controls. Among the 5 candidates, TOMM22 and RPS27A were significantly upregulated in RP, while the other three showed no significant expression difference between groups (Figure 4C). Cell type‐specific mitophagy scoring further revealed that mitophagy activity was significantly reduced in epithelial cells (the primary cell type damaged during kidney stone formation) from RP tissues relative to control epithelial cells (Figure 4D). Given that RPS27A lacks a direct mitophagy‑related function, while TOMM22 is a validated mitophagy regulator, we selected TOMM22 for subsequent validation. Western blot validation in HK‐2 cells confirmed that oxalate treatment significantly upregulated TOMM22 protein expression, while HLP‐SeNPs intervention effectively reversed this upregulation (Figure S4C).

### 2.4 HLP‐SeNPs Alleviate Oxalate‐Induced Oxidative Stress and Renal Tubular Injury via Promoting Mitophagy

**FIGURE 5:**
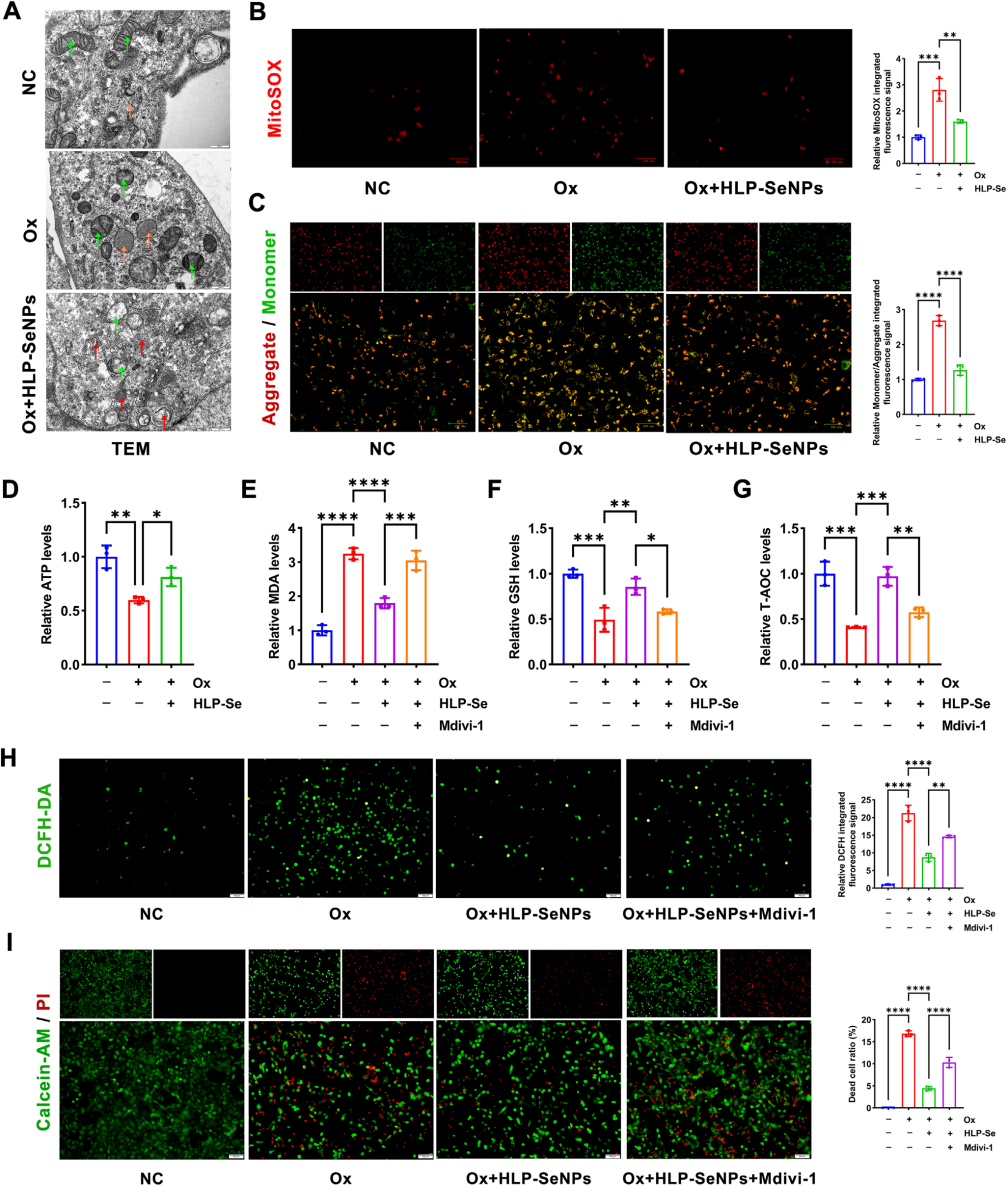
HLP‐SeNPs protect HK‐2 cells from oxalate‐induced mitochondrial damage and oxidative stress in a mitophagy‐dependent manner. (A) TEM microstructure of HK‐2 cells showing mitochondria (green arrows), endoplasmic reticulum (orange arrows), and autophagosomes (red arrows). (B) Representative MitoSOX staining images and semi‐quantitative analysis of mitochondrial superoxide level. (C) Representative JC‐1 staining images and semi‐quantitative analysis of mitochondrial membrane potential. (D) Quantification of relative ATP level. (E‐G) Quantification of MDA (E), GSH (F), and T‐AOC (G) levels with or without Mdivi‐1 co‐treatment. (H) Representative DCFH‐DA staining images for total ROS and semi‐quantitative analysis. (I) Representative Calcein‐AM/PI double staining (live cells: green, dead cells: red) and semi‐quantitative analysis of dead cell ratio. Data are presented as mean ± SD. *p* < 0.05, ^*^ *p* < 0.01, ^**^ *p* < 0.001, ^***^ *p* < 0.0001.

To explore the mechanism underlying the nephroprotective effect of HLP‐SeNPs, we first examined mitophagy activation and oxidative stress status in Ox‐treated human renal tubular epithelial HK‐2 cells. TEM of HK‐2 cells revealed distinct ultrastructural differences among experimental groups. Control group cells showed abundant and intact mitochondria distributed throughout the cytoplasm. In contrast, cells treated with Ox demonstrated pathological changes, including swollen mitochondria, disappearance of mitochondrial cristae, reduced matrix electron density, and endoplasmic reticulum expansion. Cells in the HLP‐SeNPs treatment group exhibited numerous autophagic vesicles (Figure 5A). MitoSOX fluorescence analysis indicated that oxalate significantly elevated mitochondrial superoxide levels, a hallmark of oxidative stress, whereas treatment with HLP‐SeNPs effectively suppressed this increase (Figure 5B). JC‐1 fluorescent staining further demonstrated that HLP‐SeNPs restored the mitochondrial membrane potential that was compromised by Ox treatment, as shown by the significantly increased ratio of JC‐1 aggregates to monomers (Figure 5C). ATP measurement also validated that HLP‐SeNPs reversed oxalate‐induced energy metabolism dysfunction (Figure 5D).

To confirm that the protective effect of HLP‐SeNPs is mitophagy‐dependent, we co‐treated cells with the specific mitophagy inhibitor Mdivi‐1. As expected, Mdivi‐1 abolished all the beneficial effects of HLP‐SeNPs. HLP‐SeNPs reduced oxalate‐induced lipid peroxidation and restored the decreased antioxidant indicators, all these effects were significantly reversed after mitophagy inhibition (Figure 5E–G). DCFH‐DA staining for total cellular ROS further confirmed that the ROS‐scavenging capacity of HLP‐SeNPs was eliminated by Mdivi‐1 (Figure 5H), and Calcein‐AM/PI double staining showed that Mdivi‐1 reversed the protective effect of HLP‐SeNPs against oxalate‐induced cell death (Figure 5I).

**FIGURE 6:**
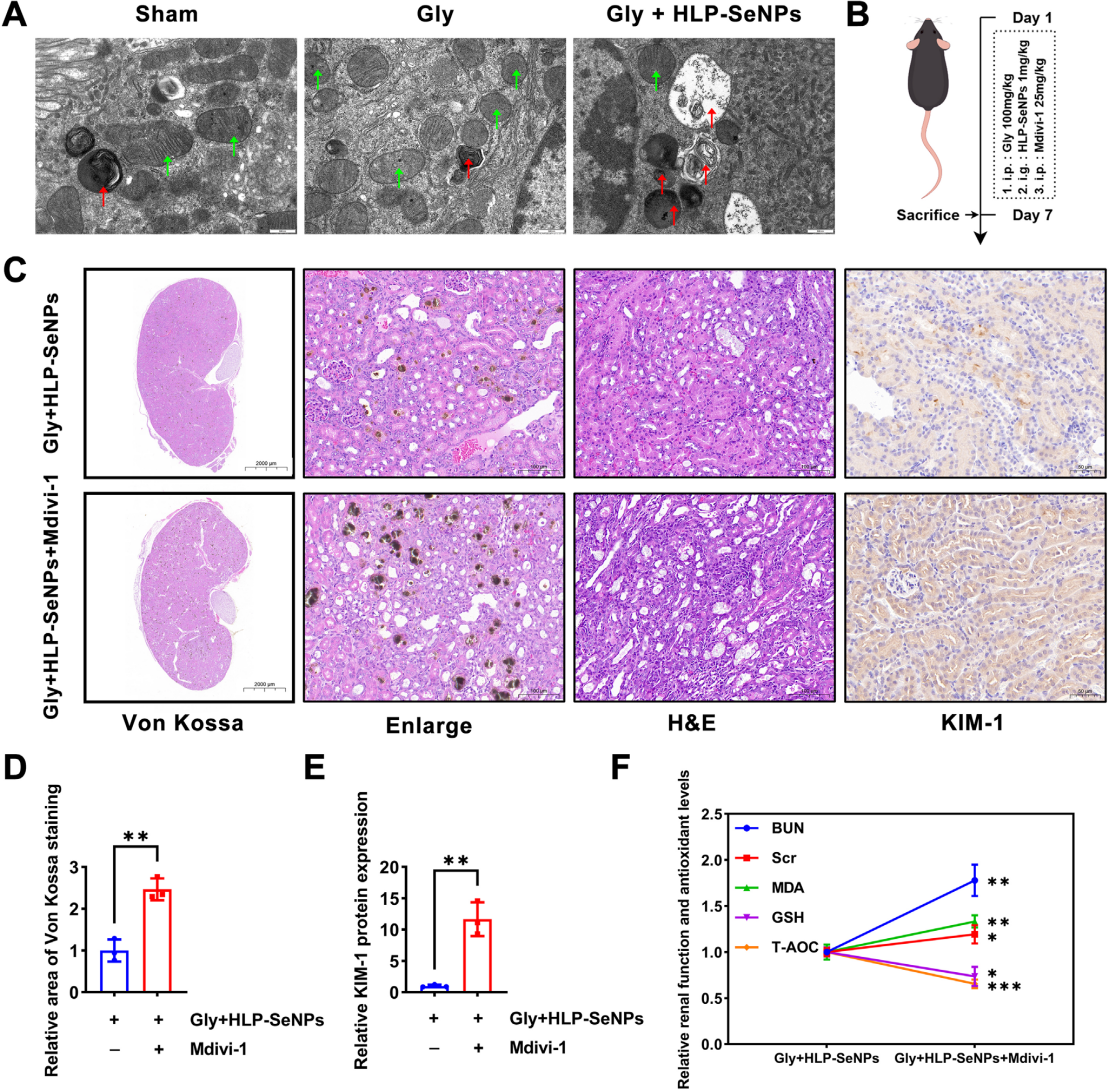
The renoprotective effect of HLP‐SeNPs in calcium oxalate nephrolithiasis mice is dependent on mitophagy activation. (A)TEM microstructure observations of mouse kidney tissues showing mitochondria (green arrows) and autophagosomes (red arrows). (B) Schematic diagram of the in vivo experimental procedure. (C) Representative images of Von Kossa staining, H&E staining, and KIM‐1 immunohistochemical staining. (D) Semi‐quantification of relative crystal deposition area based on Von Kossa staining. (E) Semi‐quantitative analysis of relative KIM‐1 protein expression level. (F) Quantification of renal function indexes (BUN, Scr) and oxidative stress indexes (MDA, GSH, T‐AOC) in each group. Data are presented as mean ± SD. ^*^ *p* < 0.05, ^**^ *p* < 0.01, ^***^ *p* < 0.001.

These findings were further confirmed in an in vivo animal model. TEM analysis of kidney sections revealed that mitochondria in the Sham group maintained a normal structure along with sparse autophagic vesicles. However, in the Gly group, mitochondria exhibited extensive loss of cristae. In contrast, renal tissues from the HLP‐SeNPs‐treatment group displayed significantly increased autophagic vesicles (Figure 6A). To evaluate the functional role of mitophagy in renal protection, mice receiving HLP‐SeNPs treatment were co‐treated with Mdivi‐1 to inhibit mitophagy (Figure 6B). Histological analysis showed that mitophagy inhibition significantly increased calcium oxalate crystal deposition, and exacerbated renal structural injury (Figure 6C). Semi‐quantitative analysis confirmed that both the relative area of crystal deposition and the expression of renal injury marker KIM‐1 were significantly higher in the Mdivi‐1 co‐treatment group than in the HLP‐SeNPs monotherapy group Figure 6D,E). Consistently, Mdivi‐1 co‐treatment significantly elevated the levels of renal function markers, increased MDA level, and decreased the activity of antioxidant indicators GSH and T‐AOC, demonstrating that mitophagy inhibition abrogated the renoprotective and antioxidant effects of HLP‐SeNPs (Figure 6F).

Together, the in vitro and in vivo data strongly suggest that HLP‐SeNPs alleviate oxalate‐induced oxidative stress and kidney injury primarily through the activation of mitophagy.

### 2.5 HLP‐SeNPs Alleviate Oxalate‐Induced Renal Tubular Injury by Downregulating TOMM22 to Activate PINK1‐Parkin‐Mediated Mitophagy

**FIGURE 7:**
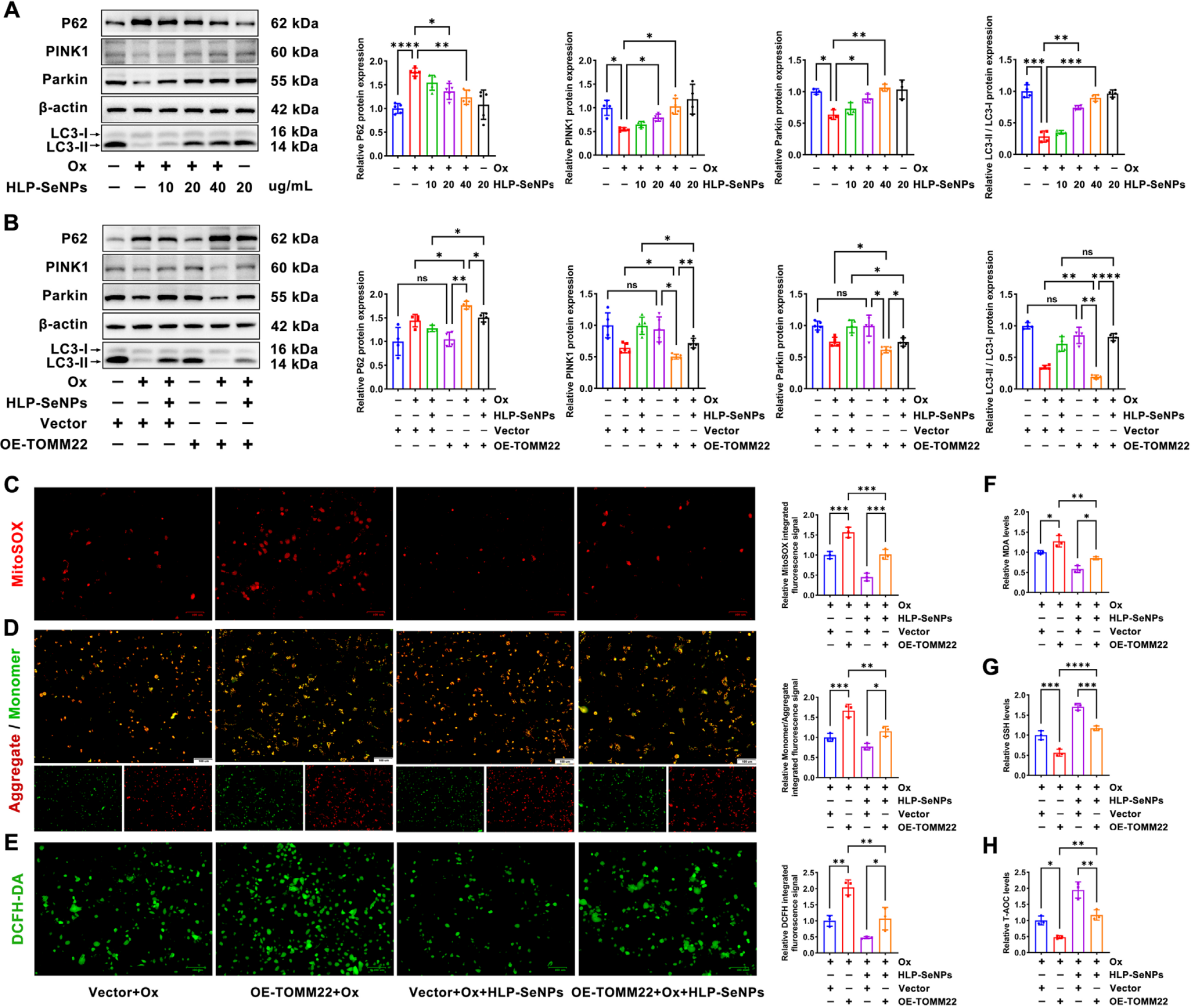
TOMM22 overexpression reverses the protective effect of HLP‐SeNPs against oxalate ‐induced injury and inhibits PINK1‐Parkin mitophagy activation. (A) Western blotting and semi‐quantitative analysis of PINK1‐Parkin pathway proteins after treatment with different concentrations of HLP‐SeNPs in oxalate‐induced HK‐2 cells. (B) Western blotting and semi‐quantitative analysis of PINK1‐Parkin pathway proteins in Vector control and OE‐TOMM22 cells under different treatments. (C) Representative MitoSOX staining images and semi‐quantitative analysis of mitochondrial superoxide level. (D) Representative JC‐1 staining images and semi‐quantitative analysis of mitochondrial membrane potential. (E) Representative DCFH‐DA staining images for total ROS and semi‐quantitative analysis. (F‐H) Quantification of MDA (F), GSH (G), and T‐AOC (H) levels in each group. Data are presented as mean ± SD. ^*^ *P* < 0.05, ^**^ *P* < 0.01, ^***^ *P* < 0.001, ^****^ *P* < 0.0001, ns: no significance.

To verify whether TOMM22 acts as a critical mediator of the protective effect of HLP‐SeNPs against oxalate‐induced injury in HK‐2 cells, we generated a HK‐2 cell line with stable overexpression of TOMM22 (OE‐TOMM22, Figure S5A). We first confirmed the dose‐dependent regulatory effect of HLP‐SeNPs on PINK1‐Parkin mitophagy in Ox‐treated HK‐2 cells. HLP‐SeNPs treatment upregulated PINK1 and Parkin protein expression, downregulated p62, and increased the LC3‐II/I ratio in a concentration‐dependent manner (Figure 7A). We next detected the effect of TOMM22 overexpression on mitophagy and cell injury under different treatments. Under oxalate stress, TOMM22 overexpression remarkably inhibited the activation of PINK1‐Parkin pathway induced by HLP‐SeNPs. Compared with vector cells treated with HLP‐SeNPs, OE‐TOMM22 cells showed decreased expression of PINK1 and Parkin, upregulated p62, and reduced LC3‐II/I ratio, indicating that overexpression of TOMM22 blocks the regulatory effect of HLP‐SeNPs on PINK1‐Parkin mitophagy (Figure 7B). We further evaluated phenotypic changes of cell injury. MitoSOX staining showed that TOMM22 overexpression significantly increased mitochondrial superoxide accumulation under oxalate stress, and completely reversed the inhibitory effect of HLP‐SeNPs on mitochondrial ROS production (Figure 7C). Consistent with the MitoSOX results, JC‐1 staining for mitochondrial membrane potential demonstrated that TOMM22 overexpression exacerbated oxalate‐induced mitochondrial membrane potential loss, reversing the protective effect of HLP‐SeNPs on mitochondrial function (Figure 7D). We further quantified oxidative stress markers in each group. Compared with the vector group, OE‐TOMM22 aggravated oxalate‐induced oxidative stress, manifested as elevated total cellular ROS and MDA levels, and decreased reduced GSH and T‐AOC levels. Notably, all the antioxidative and cytoprotective effects of HLP‐SeNPs were suppressed by TOMM22 overexpression (Figure 7E–H).

**FIGURE 8:**
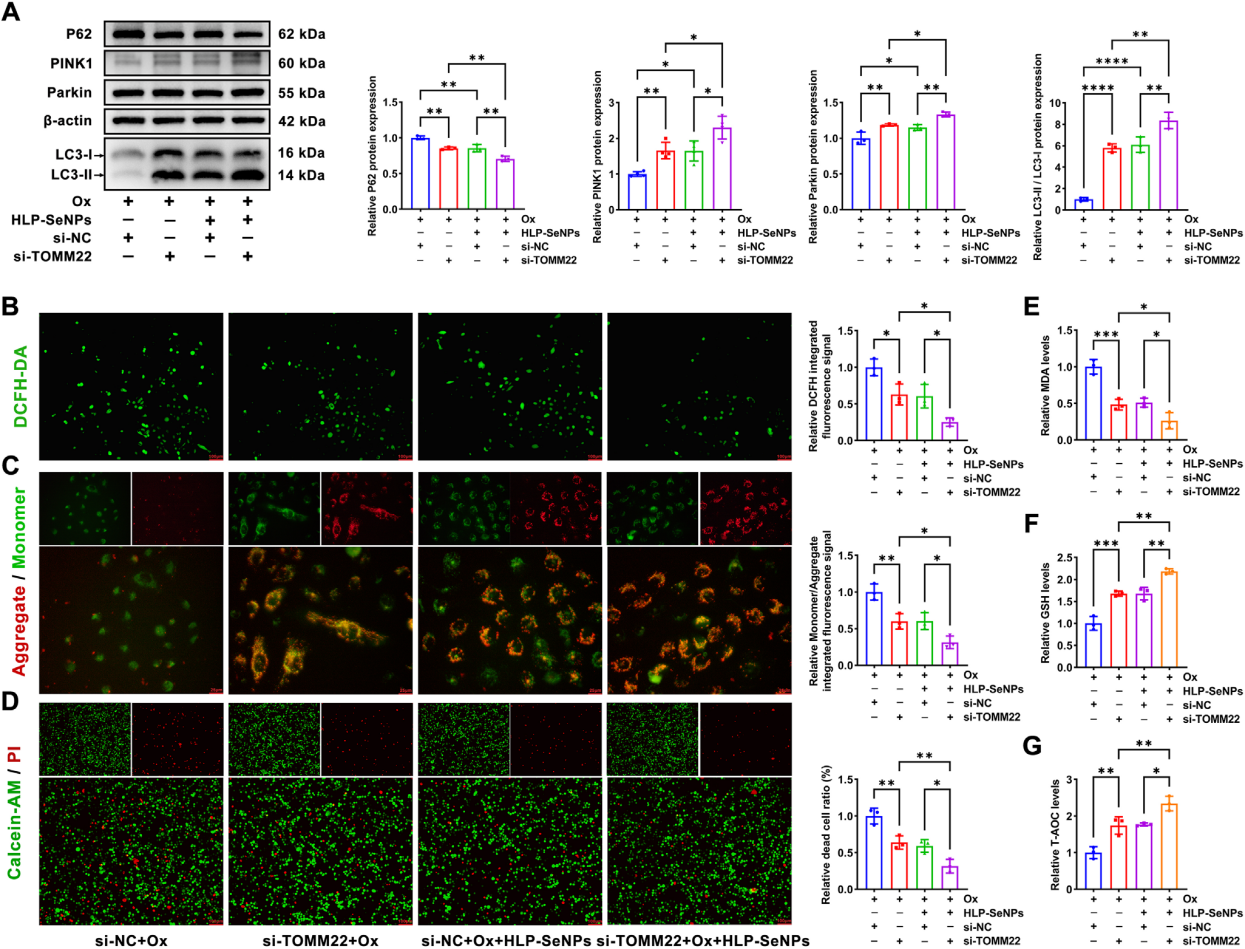
Knockdown of TOMM22 enhances the protective effect of HLP‐SeNPs against oxalate‐induced injury and promotes PINK1‐Parkin mitophagy activation. (A) Western blotting and semi‐quantitative analysis of PINK1‐Parkin pathway proteins in si‐NC control and si‐TOMM22 knockdown HK‐2 cells under different treatments. (B) Representative DCFH‐DA staining images for total ROS detection and corresponding semi‐quantitative analysis. (C) Representative JC‐1 staining images and semi‐quantitative analysis of mitochondrial membrane potential. (D) Representative Calcein‐AM/PI double staining and semi‐quantitative analysis of dead cell ratio. (E‐G) Quantification of MDA (E), GSH (F), and T‐AOC (G) levels in each group. Data are presented as mean ± SD. ^*^ *P* < 0.05, ^**^ *P* < 0.01, ^***^ *P* < 0.001, ^****^ *P* < 0.0001.

To further confirm the role of TOMM22 in the protective effect of HLP‐SeNPs, we performed reverse validation by knocking down endogenous TOMM22 using small interfering RNA (si‐TOMM22, Figure S5B) in HK‐2 cells. Consistent with the overexpression results, western blotting showed that under oxalate stress, TOMM22 knockdown further promoted the activation of PINK1‐Parkin pathway induced by HLP‐SeNPs. Compared with si‐NC, si‐TOMM22 combined with HLP‐SeNPs treatment further downregulated p62 protein, upregulated PINK1 and Parkin, and increased the LC3‐II/I ratio (Figure 8A). We further detected changes of oxidative stress and cell viability. TOMM22 knockdown significantly reduced oxalate‐induced total ROS production, decreased MDA level, increased GSH and T‐AOC levels, and reduced the proportion of dead cells, which further enhanced the antioxidative and cytoprotective effects of HLP‐SeNPs (Figure 8B–G). Collectively, gain‐ and loss‐of‐function experiments confirmed that TOMM22 is a key functional target of HLP‐SeNPs for regulating PINK1‐Parkin mitophagy and alleviating oxalate‐induced renal tubular cell injury.

**FIGURE 9:**
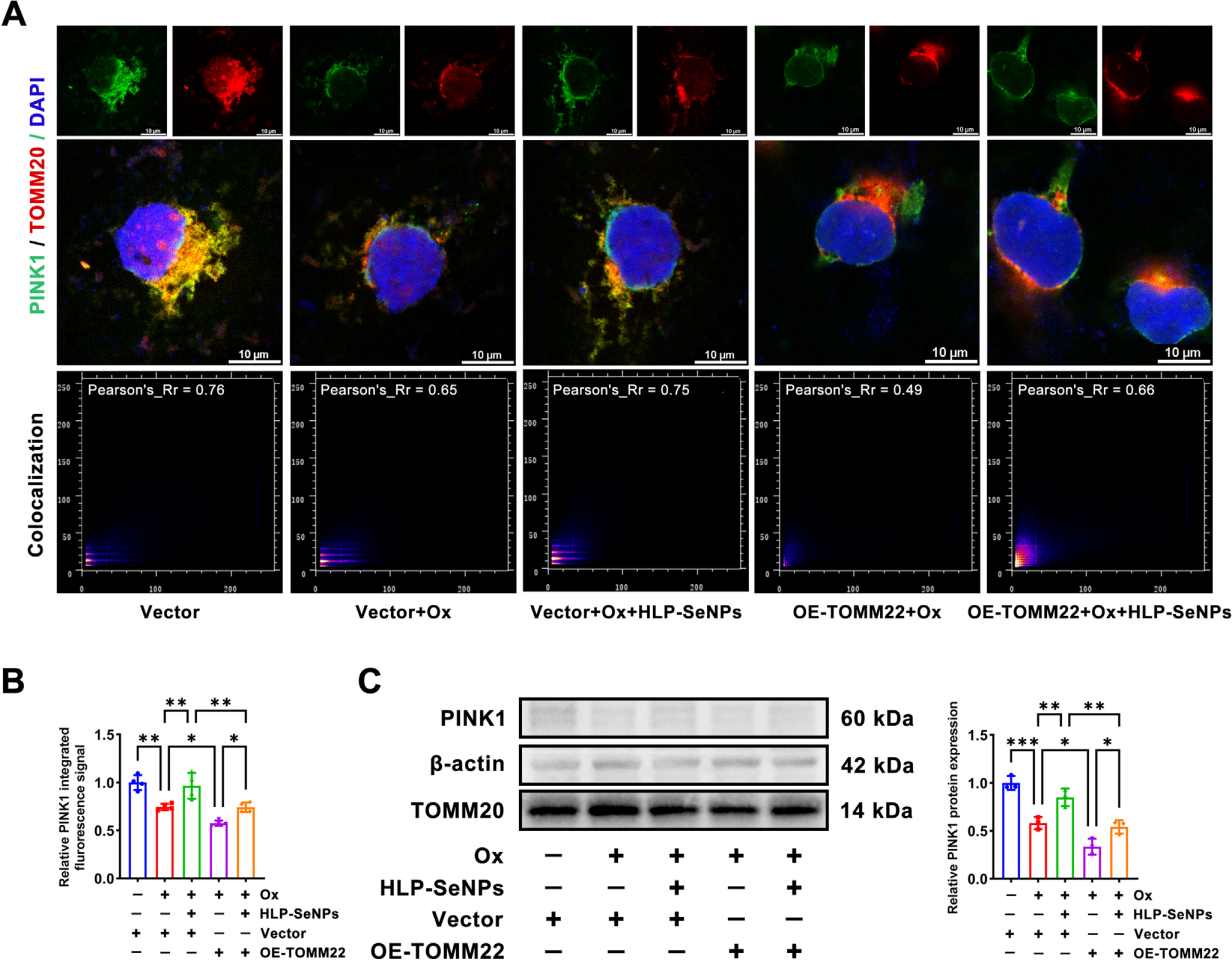
HLP‐SeNPs promotes PINK1 accumulation on the mitochondrial outer membrane by downregulating TOMM22. (A) Representative immunofluorescence images and colocalization analysis of PINK1 (green) and TOMM20 (red, mitochondrial outer membrane marker); DAPI (blue) stains the nucleus. Pearson's correlation coefficient (Pearson's Rr) for colocalization is shown for each group. (B) Semi‐quantitative analysis of PINK1 integrated fluorescence intensity in each group. (C) Western blotting of PINK1 protein in isolated mitochondrial fractions and corresponding semi‐quantitative analysis of relative PINK1 expression. Data are presented as mean ± SD. ^*^ *P* < 0.05, ^**^ *P* < 0.01, ^***^ *P* < 0.001.

TOMM22 is a core component of the mitochondrial outer membrane translocase complex, which is responsible for importing cytoplasmically synthesized proteins including PINK1 into mitochondria. Stabilization and accumulation of PINK1 on the mitochondrial outer membrane is the initial trigger of PINK1‐Parkin mitophagy [31, 32]. Based on the above results, we hypothesized that HLP‐SeNPs downregulates TOMM22 to reduce the import of PINK1 into mitochondria, thereby promoting PINK1 retention on the mitochondrial outer membrane to initiate mitophagy. To test this hypothesis, we performed immunofluorescence co‐localization analysis of PINK1 and TOMM20 (a mitochondrial outer membrane marker) in different treatment groups. The results showed that oxalate stress decreased PINK1‐TOMM20 colocalization, which was partially restored by HLP‐SeNPs but completely abolished by TOMM22 overexpression (Figure 9A,B). To further verify PINK1 accumulation in mitochondria, we isolated mitochondrial proteins and detected PINK1 levels by western blotting. Consistent with the immunofluorescence results, oxalate stress decreased mitochondrial PINK1 accumulation, HLP‐SeNPs significantly increased mitochondrial PINK1 content, and TOMM22 overexpression reversed the restorative effect of HLP‐SeNPs on mitochondrial PINK1 (Figure 9C). These results directly confirmed that TOMM22 mediates the translocation of PINK1 into mitochondria, and downregulation of TOMM22 by HLP‐SeNPs promotes PINK1 retention on the mitochondrial outer membrane to initiate mitophagy.

**FIGURE 10:**
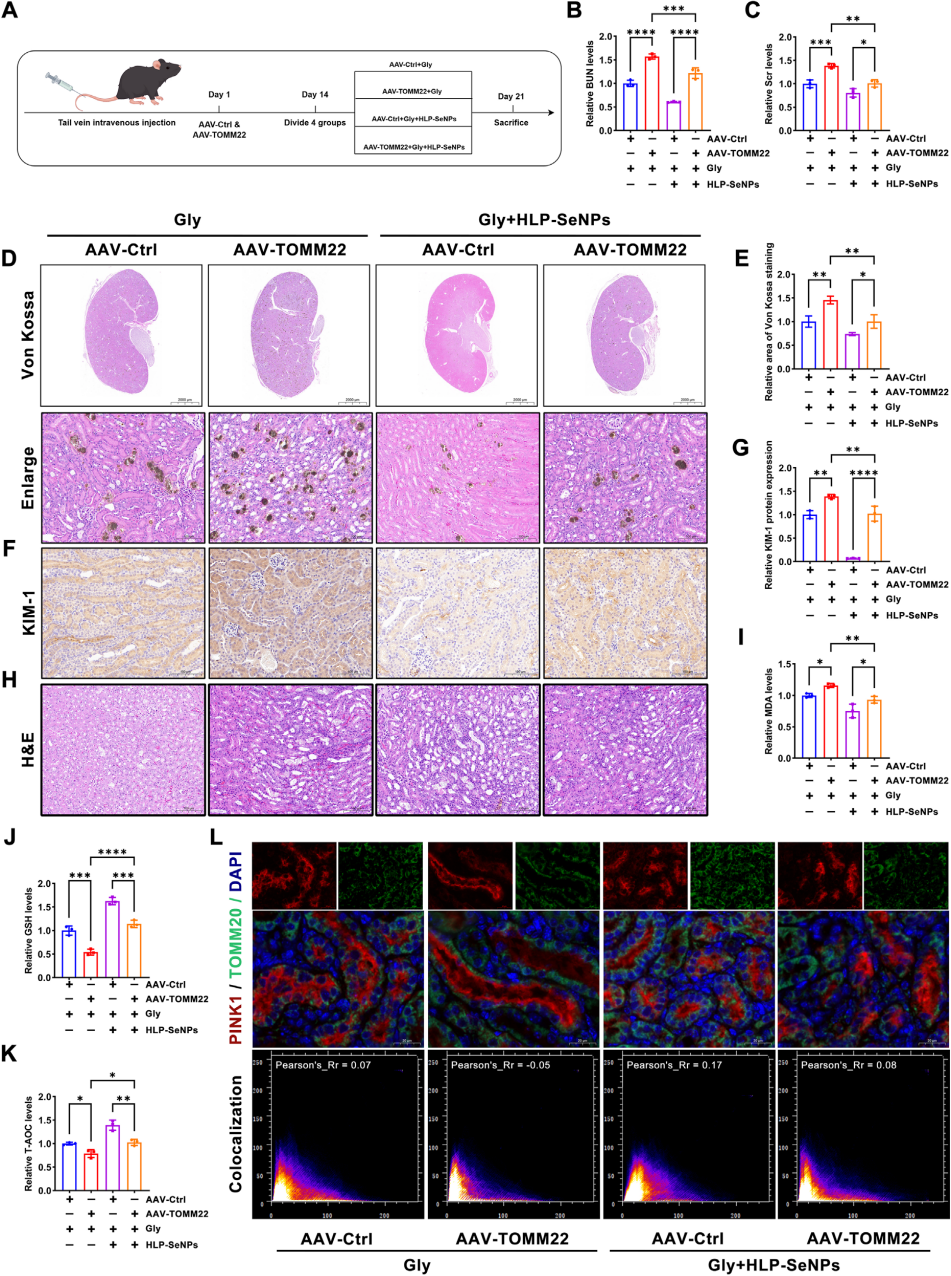
Renal‐targeted overexpression of TOMM22 attenuates the protective effects of HLP‐SeNPs against glyoxylate‐induced renal injury in mice. (A) Schematic diagram of in vivo animal experiment design; (B‐C) Quantification of relative BUN and Scr levels for renal function assessment; (D) Representative Von Kossa staining images of CaOx crystals in mouse kidney tissues; (E) Semi‐quantitative analysis of Von Kossa positive crystal area; (F) Representative images of KIM‐1 immunohistochemical staining in kidney tissues; (G) Semi‐quantitative analysis of relative KIM‐1 protein expression; (H) Representative H&E staining images of renal histological injury; (I‐K) Quantification of relative MDA (I), GSH (J), and T‐AOC (K) level in renal tissues; (L) Representative immunofluorescence images and co‐localization analysis of PINK1 (red) and TOMM20 (green) in renal tissues. Data are presented as mean ± SD. ^*^ *P* < 0.05, ^**^ *P* < 0.01, ^***^ *P* < 0.001, ^****^ *P* < 0.0001.

To further confirm the role of TOMM22 in HLP‐SeNPs‐mediated anti‐nephrolithiasis effect in vivo, we constructed adeno‐associated virus (AAV)‐mediated TOMM22 overexpression mouse model (Figure S5C). The schematic of in vivo experimental design is shown in Figure 10A. Renal function detection showed that BUN and Scr levels were significantly elevated in TOMM22‐overexpressing mice after glyoxylate induction, and the renal function protective effect of HLP‐SeNPs was significantly impaired (Figure 10B,C). Von Kossa staining showed that TOMM22 overexpression significantly increased CaOx crystal deposition in mouse kidneys, and the crystal‐clearing ability of HLP‐SeNPs was markedly abrogated (Figure 10D,E). H&E staining revealed more severe renal histological damage in the TOMM22 overexpression group (Figure 10H), and immunohistochemical staining of renal injury marker KIM‐1 further confirmed that TOMM22 overexpression aggravated glyoxylate‐induced renal injury, and offset the protective effect of HLP‐SeNPs (Figure 10F,G). For oxidative stress detection, TOMM22 overexpression significantly increased MDA level, and decreased GSH and T‐AOC levels in renal tissues, which completely eliminated the antioxidant effect of HLP‐SeNPs (Figure 10I–K). Immunofluorescence co‐localization analysis further verified that TOMM22 overexpression reduced the co‐localization level of PINK1 and mitochondrial marker TOMM20 in vivo, which was consistent with our mechanistic hypothesis (Figure 10L).

Overall, these findings indicate that HLP‐SeNPs alleviate oxalate‐induced kidney injury and oxidative stress by modulating TOMM22 to activate PINK1‐Parkin‐mediated mitophagy.

## 3 Discussion

**FIGURE 11:**
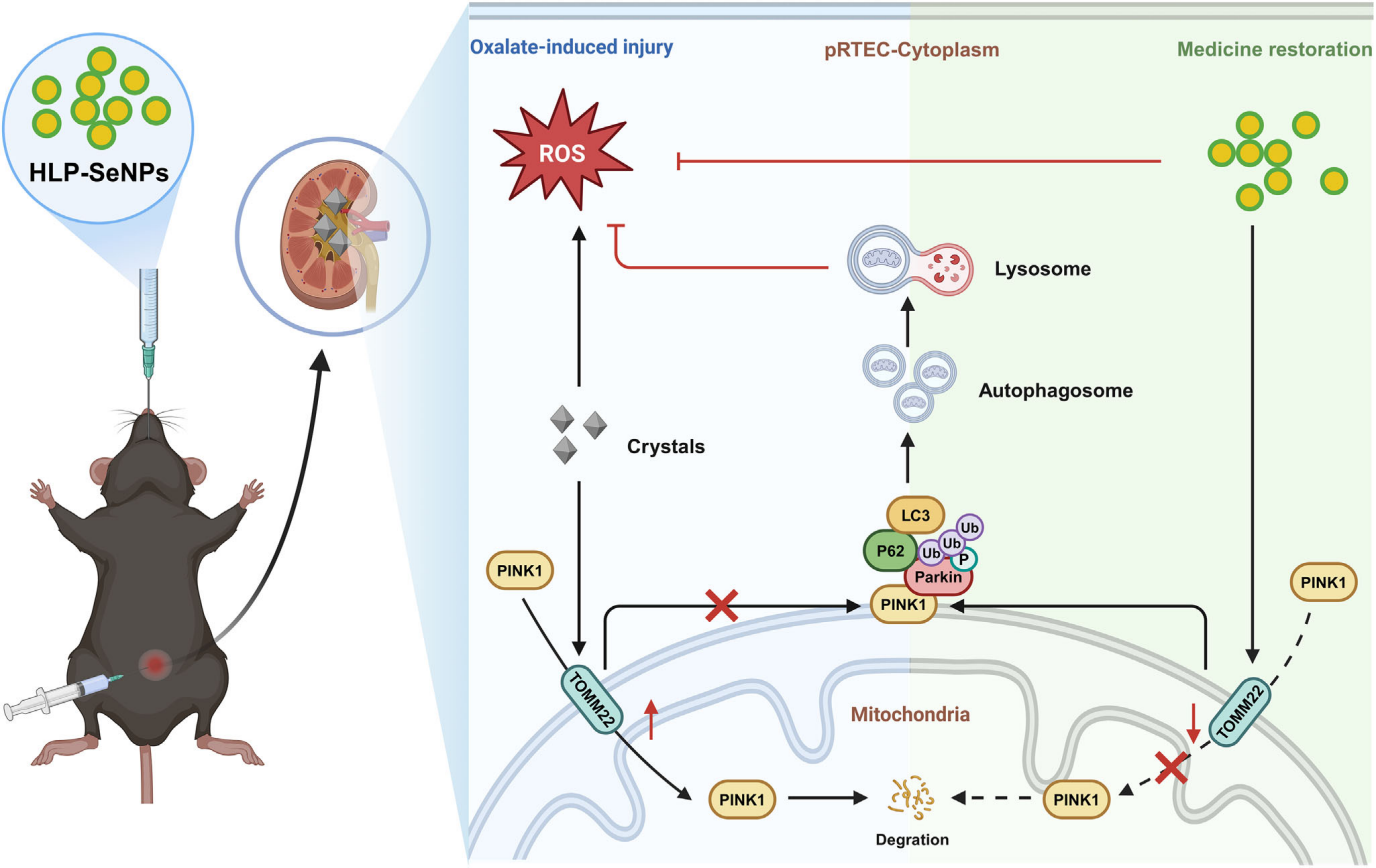
HLP‑SeNPs activate PINK1‐Parkin‐Mediated mitophagy via TOMM22 downregulation to alleviate oxidative stress in kidney stone disease. Oxalate crystals induce excessive ROS production in RTECs. At the same time, oxalate crystals cause high expression of TOMM22, promoting the continuous translocation of PINK1 into the mitochondria, thereby preventing its stable accumulation on the outer mitochondrial membrane and inhibiting the activation of PINK1/Parkin‐mediated mitophagy. This leads to the inability to clear damaged mitochondria, further exacerbating ROS accumulation, and ultimately results in oxalate‐induced cellular and kidney injury. HLP‐SeNPs treatment restores the function of the PINK1/Parkin mitophagy pathway. It downregulates TOMM22 expression, reduces the import of PINK1 into mitochondria, and promotes its retention on the outer mitochondrial membrane. This action re‐initiates PINK1/Parkin‐mediated mitophagy, enabling damaged mitochondria to be engulfed by autophagosomes and excessive ROS to be cleared, thereby achieving a restorative effect of HLP‐SeNPs against oxalate‐induced kidney injury.

KSD has become a significant global public health burden, with oxidative stress‐induced RTEC injury identified as a key pathogenic mechanism. However, current therapeutic strategies rely predominantly on surgical interventions, and effective drug‐based approaches remain unavailable [33]. In this study, we developed SeNPs modified with HLP derived from *Lysimachia christinae*. For the first time, we systematically elucidate their mechanism of action, demonstrating that HLP‐SeNPs alleviate renal injury and oxidative stress by specifically downregulating TOMM22 to activate PINK1‐Parkin‐mediated mitophagy (Figure 11). By addressing the lack of mitophagy‐targeted therapies for nephrolithiasis, this study introduces a novel and promising avenue for nanomedicine development in kidney stone treatment.

The design of HLP‐SeNPs carefully integrates both the physicochemical properties of nanodrugs and their potential biological impacts. Shifts in the characteristic peaks of ─OH and C─O─H bonds observed in FTIR spectroscopy confirm successful covalent modification between HLP and SeNPs. The encapsulation of SeNPs with HLP significantly enhances their stability in SGF and SIF, which is crucial for the efficacy and bioavailability of orally administered drugs. Moreover, as demonstrated by T‐AOC assays, HLP‐SeNPs exhibit markedly superior antioxidant capacity compared to SeNPs, underscoring a strong synergistic effect between HLPs and SeNPs. These optimized physicochemical properties provide a robust foundation for the therapeutic performance of HLP‐SeNPs, which was further validated by their substantial efficacy in vivo, thus reinforcing the rationality of their design.

Oral administration of HLP‐SeNPs resulted in a pronounced reduction in renal CaOx crystal deposition and a significant attenuation of renal tubular injury. Additionally, HLP‐SeNPs markedly improved oxidative stress‐related biochemical indices, demonstrating their capability to reverse oxalate‐induced oxidative damage effectively. Biodistribution analysis confirms the renal‐targeting properties of HLP‐SeNPs, while their rapid metabolism minimizes the risks of cumulative toxicity, reflecting pharmaceutically desirable pharmacokinetic characteristics for nanodrugs.

*Lysimachia christinae*, a classic herb in traditional Chinese medicine known for its diuretic and detoxifying properties, has been used to treat nephrolithiasis for centuries. Recent pharmacological studies have confirmed its multi‐target effects, such as mitigating oxidative stress and regulating gut microbiota metabolism, that effectively prevent stone formation [34, 35]. Building on this rich foundation, our study applies HLP to modify SeNPs for the first time, imparting the nanoparticles with additional antioxidant and stability‐enhancing properties. Moreover, bioinformatics analysis based on Astral‐DIA proteomics established the mechanistic link between HLP‐SeNPs and mitophagy for the first time. This study identifies TOMM22 as a pivotal regulatory node mediating the protective effects of HLP‐SeNPs. The kidney, as a high‐energy metabolic organ, relies heavily on mitochondrial quality control for proper function [36]. When mitophagy is impaired, oxalate‐induced mitochondrial damage exacerbates ROS accumulation and cellular injury. Intervention with HLP‐SeNPs restores this balance by reactivating the mitophagic clearance of damaged mitochondria, addressing the oxidative stress induced by oxalate at its root cause. Importantly, this study revealed that the therapeutic effects of HLP‐SeNPs extend beyond ROS scavenging. HLP‐SeNPs downregulate TOMM22, thereby enhancing the efficiency of PINK1 translocation into mitochondria to activate PINK1‐Parkin‐mediated mitophagy. This precise targeting and mechanistic clarification not only deepen our understanding of nephrolithiasis pathophysiology but also suggest promising new avenues for developing precision medicine interventions that leverage mitophagy as a therapeutic target.

TOMM22, a core subunit of the translocase of the outer mitochondrial membrane complex, plays a critical role in the transmembrane import of nuclear‐encoded mitochondrial proteins [37]. Previous study has shown that overexpression of TOMM22 disrupts mitochondrial protein import and function, contributing to the pathogenesis of pancreatic cancer [38]. Bertolin et al. further identified that the TOMM complex, with TOMM22 and TOMM40 as core subunits, serves as a molecular switch for PINK1‐Parkin‐dependent mitophagy [39]. Our study demonstrates that HLP‐SeNPs downregulate TOMM22 expression, promoting PINK1 accumulation on the outer mitochondrial membrane. This, in turn, activates PINK1‐Parkin‐mediated mitophagy and attenuates oxalate‐induced renal tubular damage and oxidative stress. A particularly intriguing observation is that TOMM22 overexpression does not significantly affect mitophagy levels under baseline physiological conditions. However, under oxalate‐induced stress, TOMM22 overexpression appears to markedly suppress mitophagy activation. This suggests that TOMM22's involvement in mitophagy is contingent on the presence of mitochondrial damage signals. Based on these findings, we propose a plausible mechanism: under oxalate‐induced pathological conditions, excessive TOMM22 expression may lead to aberrant translocation of PINK1 into the mitochondrial matrix, thereby reducing its effective accumulation on the outer membrane. The diminished presence of PINK1 on the mitochondrial outer membrane consequently weakens Parkin recruitment and activation, ultimately impairing mitophagy. Future studies should aim to quantify phosphorylated PINK1 levels and analyze mitochondrial ultrastructure under varying TOMM22 expression and oxalate stress conditions to further validate and refine this hypothesis.

From the perspective of clinical translation, the findings of this study have significant therapeutic potential. As a high‐energy metabolic organ, the kidney is particularly vulnerable to mitochondrial dysfunction, which is not only implicated in nephrolithiasis but is also a common pathological basis for acute kidney injury, chronic kidney disease, and diabetic nephropathy [40, 41, 42]. The mechanism of HLP‐SeNPs, which alleviates oxidative stress by activating mitophagy, could theoretically be applied to these conditions as well. Additionally, the preparation and evaluation system established for polysaccharide‐modified nanoparticles in this study could be extended to other natural polysaccharides and functional nanomaterials, offering a pioneering framework for the development of hybridized herbal‐nano pharmaceuticals.

Despite the breakthroughs achieved, this study has several limitations. First, the glyoxylate‐induced CaOx model, while rapid and easily controlled, does not fully replicate the mechanisms of CaOx stone formation or the pathophysiology of other types of kidney stones. Future studies should aim to validate the therapeutic efficacy of HLP‐SeNPs across multiple kidney stone models to establish their broader applicability. Second, although we identified downregulation of TOMM22 as a critical action of HLP‐SeNPs, the precise regulatory mechanism remains unclear. Whether this involves direct interactions between HLP or SeNP components and TOMM22, or an upstream signaling pathway, warrants further investigation. Future studies could employ co‐immunoprecipitation combined with mass spectrometry to elucidate the interaction between HLP and TOMM22. Lastly, while preliminary biodistribution data using Cy5 fluorescence tracing provides insights into HLP‐SeNP targeting, more comprehensive studies on the pharmacokinetics, long‐term toxicity, and repeated‐dose toxicity of HLP‐SeNPs are needed for a complete safety evaluation.

## 4 Conclusion

This study developed a novel nanomaterial of HLP‐SeNPs and, for the first time, demonstrated their mechanism of action in alleviating oxidative stress‐induced injury associated with kidney stones. Through the downregulation of TOMM22, HLP‐SeNPs activate PINK1‐Parkin‐mediated mitophagy, enhancing mitochondrial homeostasis. The modification with HLP significantly improves SeNP stability and synergistically boosts their antioxidant capacity. HLP‐SeNPs significantly reduce CaOx crystal deposition and renal tubular injury both in vitro and in vivo, exhibiting excellent renal targeting and safety. Overall, this study highlights a promising strategy for mitophagy‐targeted therapies in nephrolithiasis treatment and demonstrates the potential of integrating traditional Chinese medicine with nanotechnology.

## 5 Method

### 5.1 Preparation and Characterization of HLP‐SeNPs

HLP (TDT103, Winherb, China) solution (2 mg/mL), sodium selenite solution (10 mM, 214485, MERCK, Germany), and ascorbic acid solution (40 mM, HY‐B0166, MCE, USA) were prepared using PBS buffer (G4202, Servicebio, China) as the solvent. The HLP solution and sodium selenite solution were mixed vigorously at a 1:4 (v/v) ratio for 30 min, followed by the addition of an equal volume of ascorbic acid solution under continuous stirring at 37°C for 24 h. After the reaction, the supernatant was discarded by centrifugation, and the precipitate was resuspended in an appropriate volume of PBS buffer, then washed three times by centrifugation at 12 000 rpm for 10 min each. This procedure yielded HLP‐SeNPs, which were stored at 4°C. SeNPs were prepared using identical steps, replacing the HLP solution with distilled water. Cy5‐labeled HLP‐SeNPs (Cy5‐HLP‐SeNPs) were prepared by slowly adding 0.7 mL Cy5 solution (1 mg/mL, HY‐D0821, MCE) in the dark before introducing the ascorbic acid solution; subsequent steps were unchanged.

The morphology and structure of HLP‐SeNPs were analyzed using transmission electron microscopy (TEM, JEOL, Japan) coupled with energy dispersive X‐ray spectroscopy (EDX) to determine elemental composition and distribution. The hydrodynamic particle size distribution and zeta potential of HLP‐SeNPs were measured using dynamic light scattering (DLS). Infrared absorption and ultraviolet‐visible spectra of HLP‐SeNPs were characterized using fourier‐transform infrared spectroscopy (FTIR, Thermo Scientific, USA) and a UV‐visible spectrophotometer (UV‐1780, Shimadzu, Japan), respectively. In vitro antioxidant capacity of HLP‐SeNPs and SeNPs was assessed using Total Antioxidant Capacity Detection Kits (T‐AOC, S0121, Beyotime, China). Additionally, the stability of HLP‐SeNPs and SeNPs was evaluated via simulated gastric fluid (SGF, A7921, Solarbio, China) and simulated intestinal fluid (SIF, A1792, Solarbio, China).

### 5.2 Animal Experiments

Male C57BL/6 mice (6‐8 weeks old and 4–5 weeks old) were purchased from Hubei Charles River Co., Ltd. The mice were housed in a controlled environment with a regular light/dark cycle, suitable temperature, and free access to food and water. After a one‐week adaptation period, the mice were randomly divided into experimental groups.

Six groups were established for the 6‐8‐week‐old mice: sham surgery (Sham, n = 3), glyoxylate‐induced kidney stone model (Gly, n = 6), selenium nanoparticle treatment (Gly + SeNPs, n = 3), HLP‐SeNPs treatment (Gly + HLP‐SeNPs, n = 6), HLP‐SeNPs‐only group (HLP‐SeNPs, n = 3), combined treatment with HLP‐SeNPs and Mdivi‐1 (Gly + HLP‐SeNPs + Mdivi‐1, n = 3), and HLP treatment group (Gly + HLP, n = 3). The 4‐5‐week‐old mice were divided into four groups: glyoxylate and control virus infection (AAV‐Ctrl + Gly, n = 3), glyoxylate and virus overexpression (AAV‐TOMM22 + Gly, n = 3), combined HLP‐SeNPs treatment with control virus infection (AAV‐Ctrl + Gly + HLP‐SeNPs, n = 3), and combined treatment with HLP‐SeNPs and virus overexpression (AAV‐TOMM22 + Gly + HLP‐SeNPs, n = 3). All treatments and glyoxylate modeling lasted for one week, with daily administration.

In the Sham group, mice were intraperitoneally injected and orally gavaged with saline. The Gly group received intraperitoneal glyoxylate (100 mg/kg/day) and saline gavage. The Gly + SeNPs, Gly + HLP‐SeNPs and Gly + HLP groups received SeNPs, HLP‐SeNPs or HLP (1 mg/kg/day) via gavage in addition to glyoxylate modeling. The HLP‐SeNPs‐only group was gavaged with HLP‐SeNPs (1 mg/kg/day) and intraperitoneally injected with saline. The Gly + HLP‐SeNPs + Mdivi‐1 group was administered glyoxylate, HLP‐SeNPs via gavage (1 mg/kg/day), and intraperitoneal Mdivi‐1 (25 mg/kg/day). Mice in the virus infection groups underwent tail vein injections of AAV‐Ctrl or AAV‐TOMM22 after one week of adaptive feeding. Virus infection was established over two weeks, followed by glyoxylate modeling and HLP‐SeNP treatment as described earlier.

Upon completion of the experiments, mice were anesthetized using 3% sodium pentobarbital (30 mg/kg via intraperitoneal injection). Blood was withdrawn via eyeball extraction, held at room temperature, then centrifuged for serum separation. Mice were placed in a supine position for midline abdominal incision, and kidney, liver, heart, spleen, and lung tissues were harvested. Tissues were either snap‐frozen in liquid nitrogen or preserved in fixative solutions.

### 5.3 Metabolism and Distribution of HLP‐SeNPs

Mice were gavaged with Cy5.5‐HLP‐SeNPs solution, and live imaging was conducted at various time points using the AniView600 in vivo imaging system. Organ‐specific imaging (heart, liver, spleen, lungs, kidney) was performed on harvested tissues.

### 5.4 Histopathology of Mouse Tissue

Fixed tissues were embedded in paraffin, sectioned, and stained with hematoxylin‐eosin (H&E) to evaluate tissue damage. Kidney sections were further subjected to Von Kossa staining to visualize calcium crystal deposition.

### 5.5 Immunohistochemistry

Deparaffinized kidney sections underwent hydrated, antigen retrieval, blocking, and incubation with primary and secondary antibodies, followed by chromogenic staining for observation. Details of antibody usage are provided in Supplementary Table S2.

### 5.6 Evaluation of Serum Renal Function and Biochemical Indicators

Peripheral blood serum was separated via centrifugation. Kidney tissues were homogenized for protein extraction and quantification, and selected indicators were processed in an automated biochemical analyzer (Biobase, China).

### 5.7 Adeno‐Associated Virus (AAV) Packaging

For renal‐specific overexpression of TOMM22, recombinant AAV serotype 9 carrying mouse TOMM22 cDNA under the control of the kidney‐specific cadherin promoter was constructed and packaged. Plasmid vectors were constructed using seamless cloning, sequenced for verification, and purified for endotoxin‐free extraction. Plasmids and helper vectors were co‐transfected in HEK‐293T cells, and viral particles were purified from supernatants, aliquoted, and stored at ‐80°C.

### 5.8 Cell Culture

Human proximal tubular epithelial cells (HK‐2) and HEK‐293T cells, obtained from American type culture collection (ATCC), were cultured in DMEM/F12 media (G4612, Servicebio, China) supplemented with 10% fetal bovine serum (BOLG1001, Bio‐logy, China) at 37°C in a humidified 5% CO_2_ atmosphere. To establish a high‐oxalate stress model, HK‐2 cells were treated with 2 mM sodium oxalate (10020118, SinoPharm, China) with or without HLP‐SeNPs co‐incubation for 24 h. For mitophagy inhibition, cells were pretreated with 10 µM Mdivi‐1 (HY‐15886, MCE, China) for 1 h prior to oxalate stress treatments.

### 5.9 Cell Transfection

Using the EZ Trans Lentiviral Infection Kit (AC04L6, Life‐iLab, China), TOMM22 overexpression plasmid and its corresponding negative control plasmid (P81926, MiaoLingBio, China) were transfected into HEK‐293T cells to package and concentrate lentiviral particles. The resulting lentivirus was used to infect HK‐2 cells, which were subsequently screened with 5 µg/mL puromycin (G4017, Servicebio, China) to establish stable transfected cell lines. Transient transfection were performed using Lipofectamine 3000 reagent (L3000015, Invitrogen, USA) according to the manufacturer's protocol. HK‐2 cells in logarithmic phase were transfected with si‐TOMM22 small interfering RNA and negative control siRNA (GENERAL BioL, China). Subsequent assays were conducted 48 h post‐transfection.

### 5.10 Cell Viability Assay

A working solution was prepared using the Cell Counting Kit‐8 (CCK‐8, G4103, Solarbio, China). HK‐2 cells were incubated with the working solution for 2–4 h after treatment, and absorbance at 450 nm was measured using a microplate reader (Thermo Scientific, USA). Additionally, the Calcein‐AM/PI live/dead staining kit (G1707, Solarbio, China) was used to co‐incubate HK‐2 cells in the dark for 30 min. Live and dead cells were visualized under a fluorescence microscope, and cell viability was quantitatively assessed through fluorescence intensity analysis.

### 5.11 Astral‐Data Independent Acquisition (DIA) Proteomics and Bioinformatics Analysis

Samples were collected from untreated HK‐2 cells, HK‐2 cells treated with sodium oxalate for 24 h, or cells co‐treated with sodium oxalate and HLP‐SeNPs. These samples were subjected to Astral‐DIA proteomic profiling by Beijing Qinglianbio Biotechnology Co., Ltd., using the Astral platform. Briefly, proteins were extracted using SDC lysis buffer supplemented with protease inhibitors, followed by ultrasonication and centrifugation. Protein concentration was quantified, and proteins were reduced, alkylated, digested with trypsin, and acidified. Peptides were desalted using C18 columns, lyophilized, and subsequently separated via nanoLC on a ReproSil‐Pur C18 column using an 8‐min acetonitrile/formic acid gradient for DIA analysis on an Orbitrap Astral mass spectrometer. Data analysis was conducted using DIA‐NN software based on the UniProt human database. Differentially expressed proteins were identified and subjected to functional enrichment analysis. Additionally, bioinformatics was integrated with single‐cell RNA‐sequencing data retrieved from the Gene Expression Omnibus (GEO) database (accession number: GSE176155).

### 5.12 TEM

Processed HK‐2 cells and freshly collected mouse kidney tissues were fixed with electron microscopy fixation buffer (G1102, Solarbio, China) for 2–4 h. The samples were post‐fixed in osmium tetroxide, dehydrated through an ethanol gradient, embedded in resin, sectioned into ultrathin slices, stained with uranium and lead, and analyzed under a transmission electron microscope (FEI, USA). Images were acquired for further analysis.

### 5.13 Mitochondrial Function Test

Treated HK‐2 cells were incubated in the dark with MitoSOX reagent (HY‐D1055, MCE, USA) or JC‐1 mitochondrial membrane potential assay solution (C2003S, Beyotime, China) at 37°C for 30 min. Fluorescence microscopy was used to assess mitochondrial superoxide levels and membrane potential changes. ATP content in HK‐2 cells was quantified using the ATP Assay Kit (KTB1016, CheKine, China).

### 5.14 Oxidative Stress Indicator Assay

Oxidative stress markers in treated HK‐2 cells were measured by using the Malondialdehyde (MDA) Assay Kit (S0131S, Beyotime, China), Reduced Glutathione (GSH) Assay Kit (BC1175, Solarbio, China), and T‐AOC Assay Kit, which measured intracellular levels of MDA, GSH, and T‐AOC, respectively.

### 5.15 Mitochondrial Isolation and Purification

Mitochondria were isolated using a kit (C3602S, Beyotime, China). Briefly, adherent cells were collected, resuspended in Reagent A containing PMSF, incubated on ice, and homogenized. After centrifugation, the crude mitochondrial pellet was obtained, resuspended, mixed with Purification Working Solution, and centrifuged again to obtain purified mitochondria. All steps were performed at 4°C.

### 5.16 Western Blotting

After collecting HK‐2 cells, total protein was extracted, followed by quantification, separation by sodium dodecyl sulfate—polyacrylamide gel electrophoresis (SDS‐PAGE), and transfer onto polyvinylidene difluoride membranes. Membranes were blocked and incubated with target antibodies (Supplementary Table S2). Protein bands were visualized through chemiluminescence, and protein expression levels were quantified and normalized using ImageJ software.

### 5.17 Immunofluorescence

Renal paraffin sections were prepared by deparaffinization, rehydration, and antigen retrieval. HK‐2 cells were fixed and permeabilized for subsequent analysis. Both kidney sections and cells were subjected to blocking, incubation with antibodies (Supplementary Table S1), and staining with DAPI for nuclear visualization. Images were acquired using a confocal fluorescence microscope, and protein colocalization was quantified using ImageJ software.

### 5.18 Data Analysis

All experimental data are presented as mean ± standard deviation (SD). Statistical significance was evaluated using two‐tailed unpaired Student's t‐test, multiple unpaired t tests, or one‐way analysis of variance (ANOVA) in GraphPad Prism. A significance threshold of *p* < 0.05 was applied.

## Author Contributions

J.Y. contributed to conceptualization, data curation, formal analysis, methodology, visualization, and writing of the original draft. G.L. contributed to data curation, formal analysis, investigation, and methodology. W.W. contributed to data curation, formal analysis, and investigation. D.Y. contributed to investigation, methodology, and resources. J.L. contributed to methodology, resources, and validation. Y.W. contributed to resources and validation. B.W. contributed to validation and visualization. Z.M. contributed to visualization. Y.Y. contributed to investigation, funding acquisition, supervision, and writing – review and editing. Y.X. contributed to funding acquisition, project administration, supervision, and writing – review and editing. X.Y. contributed to funding acquisition, project administration, supervision, and writing – review and editing.

## Funding

This work was supported by grants from the National Natural Science Foundation of China (Nos. 82470796, 82170780, 82200852 and 82500933), and Natural Science Foundation of Hubei Province (No. 2024AFD063).

## Ethics Statement

All animal procedures were approved by the Animal Experimental Welfare and Ethics Committee of Tongji Hospital, Tongji Medical College, Huazhong University of Science and Technology (Approval No: TJH‐202410028).

## Conflicts of Interest

The authors declare no conflicts of interest.

## Supporting information




**Supporting File**: advs75784‐sup‐0001‐SuppMat.docx.

## Data Availability

The data sets used and/or analyzed during the current study are available from the corresponding author on reasonable request.
