# Supplementary material for: Herba Lysimachiae Polysaccharide‐Modified Selenium Nanoparticles Alleviate Oxidative Injury in Kidney Stones via TOMM22‐Regulated Mitophagy Activation
Source: Adv Sci (Weinh). 2026 May 19:e75784. Online ahead of print. doi: 10.1002/advs.75784 (PMC13336033; doi:10.1002/advs.75784)
Supplement: Supplementary file 1 — Supporting File: advs75784‐sup‐0001‐SuppMat.docx. [file ADVS-9999-e75784-s001.docx]

**Supplementary data**

***Herba Lysimachiae* Polysaccharide-Modified Selenium Nanoparticles Alleviate Oxidative Injury in Kidney Stones via TOMM22-Regulated Mitophagy Activation**

Junyi Yang, Guoruiyu Lyu, Wenlong Wan, Dongfeng Yuan, Jiabo Li, Yongqi Wang, Baokang Wang, Zhilong Ma, Yuanyuan Yang*, Yang Xun* and Xiao Yu**

**Table S1** **Genes corresponding to differentially expressed proteins (DEPs) in proteomics profiling and mitophagy**

| **Group** | **Genes Names** |
| --- | --- |
| **Upregulated in Oxalate but downregulated in HLP-SeNPs (46)** | *A2M/ARPC5/ATP5ME/DEGS1/DERL1/DNAJC1/DOLPP1/EARS2/EIF1B/F5/GABARAPL2/GSPT2/HSP90AB2P/HSP90AB4P/KANSL3/LITAF/MAIP1/MAPKAPK5/ME2/MMP14/MRPL30/MRPS34/MTPAP/NBAS/NDUFA3/NKX2.4/NRP1/PSMG2/PTCD1/PTP4A1/RAB11B/RBM12/RIOX2/RPL15/SCAMP2/SDHD/SIGMAR1/SLC9A6/SUPT4H1/SUPT6H/THG1L/TMEM9/TOMM22/UBE2H/UBTD2/VPS25* |
| **Downregulated in Oxalate but upregulated in HLP-SeNPs (171)** | *AGPAT1/ALPK2/ARPP19/ATOX1/ATP5F1D/ATP5PF/BRCA1/C1orf52/CALM1/CAST/CBX1/CBX5/CCN1/CD40/CD46/CD99/CDC26/CDV3/CETN2/CFDP1/CHCHD2/CHD8/CHMP4A/CLTB/CLTCL1/CMC2/CNBP/COA6/COMT/COX17/COX5A/COX5B/CPPED1/CPSF2/CRIP1/CSNK2B/CSRP1/CSTF2/DAZAP1/DIAPH2/DNAJA2/DVL3/EIF3J/EIF4EBP1/ENSA/EPCAM/ERH/ERP44/EVA1A/FABP5/FAM107B/FCRLA/FZD6/GCSH/GLUL/GNB2/GNB4/GPX1/GPX4/HDGF/HMGN1/HNRNPUL1/IGF2R/IGFBP7/INPP1/JPT1/JPT2/LACTB2/LGALS1/LMAN2/LSM1/LSM2/LSM3/LSM4/LSM6/LSM7/LSM8/MARCKS/MARCKSL1/MGAT2/MIF/MIX23/MRPS22/MT2A/MTA1/MTAP/MTPN/MYL12B/NACA/NCS1/NDUFS6/NIT2/NPC2/NT5C3B/NUCKS1/PCBD1/PCBP3/PDLIM5/PFDN4/PFDN5/PGAM1/PHF5A/PITHD1/PLBD2/PM20D2/PNPLA8/PNPO/POLR3K/PPIF/PPP1CB/PPP1R14B/PPP1R2/PPP2CA/PROCR/PTMA/PTMS/RAP1B/RBM33/RBM45/RBX1/RCN1/REXO2/RHOA/RNH1/RPIA/RPL32/RPS17/RPS27A/S100A11/S100A2/S100A4/S100A6/SBF1/SDHC/SDSL/SERF2/SIN3B/SLC35F2/SNRPD2/SOD1/STMN1/STMN2/STX17/SUMO3/TATDN1/TBC1D15/TBCA/TBPL1/TCEAL4/TLCD3A/TLCD4/TMA7/TMSB10/TMSB4X/TPI1/TPM1/TPM3/TPM4/TRIM47/TRIP6/TTC1/UQCRH/UROD/VKORC1/VPS51/YAP1/YBX1/YWHAQ/ZFAND6/ZNF593/ZYX* |
| **Mitophagy-associated genes (121)** | *AMBRA1/AMFR/ARIH1/ATF4/ATG12/ATG5/ATG9A/ATG9B/BCL2L1/BCL2L13/BECN1/BECN2/BNIP3/BNIP3L/CALCOCO2/CCZ1/CCZ1B/CITED2/CSNK2A1/CSNK2A2/CSNK2A3/CSNK2B/E2F1/EIF2AK3/EIF2S1/FIS1/FKBP8/FOXO3/FUNDC1/GABARAP/GABARAPL1/GABARAPL2/HDAC6/HIF1A/HRAS/HUWE1/JUN/KRAS/MAP1LC3A/MAP1LC3B/MAP1LC3B2/MAP1LC3BP1/MAP1LC3C/MAPK10/MAPK8/MAPK9/MARCHF5/MFN1/MFN2/MITF/mitochondrial import receptor subunit TOM7 homolog/MON1A/MON1B/MRAS/MTERF3/MTX1/MTX2/MTX3/MUL1/NBR1/NLRX1/NRAS/OPA1/OPTN/PGAM5/PHB2/PINK1/PRKN/RAB5A/RAB5B/RAB5C/RAB7A/RAB7B/RABGEF1/RELA/RHOT1/RHOT2/RPS27A/RRAS/RRAS2/SAMM50/SIAH1/SMURF1/SMURF2/SP1/SQSTM1/SRC/TAX1BP1/TBC1D15/TBC1D17/TBK1/TFE3/TFEB/TOMM20/TOMM20L/TOMM22/TOMM40/TOMM40L/TOMM5/TOMM6/TOMM7/TOMM70/TP53/TRAF2/UBA52/UBB/UBC/UBE2D2/UBE2D3/UBE2L3/UBE2N/UBE2V1/ULK1/USP15/USP30/USP8/VCP/VDAC1/VDAC2/VDAC3/VPS13C* |

**Table S2 Antibodies used in this study**

| **Antibodies** | **Source** | **Identifier** | **Application** |
| --- | --- | --- | --- |
| KIM-1 | Proteintech | 30948-1-AP | immunohistochemistry |
| AQP1 | Proteintech | 20333-1-AP | Immunofluorescence |
| TOMM20 | Servicebio | GB111481 | Immunofluorescence |
| TOMM20 | Huabio | ET1609-25 | Western blot |
| PINK1 | Servicebio | GB114934 | Immunofluorescence |
| PINK1 | Huabio | ER1706-27 | Western blot |
| Parkin | Huabio | ET1702-60 | Western blot |
| p62 | Huabio | HA721171 | Western blot |
| LC3B | Huabio | ET1701-65 | Western blot |
| HRP conjugated Goat Anti-Rabbit IgG | Servicebio | GB23303 | Western blot, immunohistochemistry |
| Cy3 conjugated Goat Anti-Rabbit IgG | Proteintech | SA00009-2 | Immunofluorescence |

**
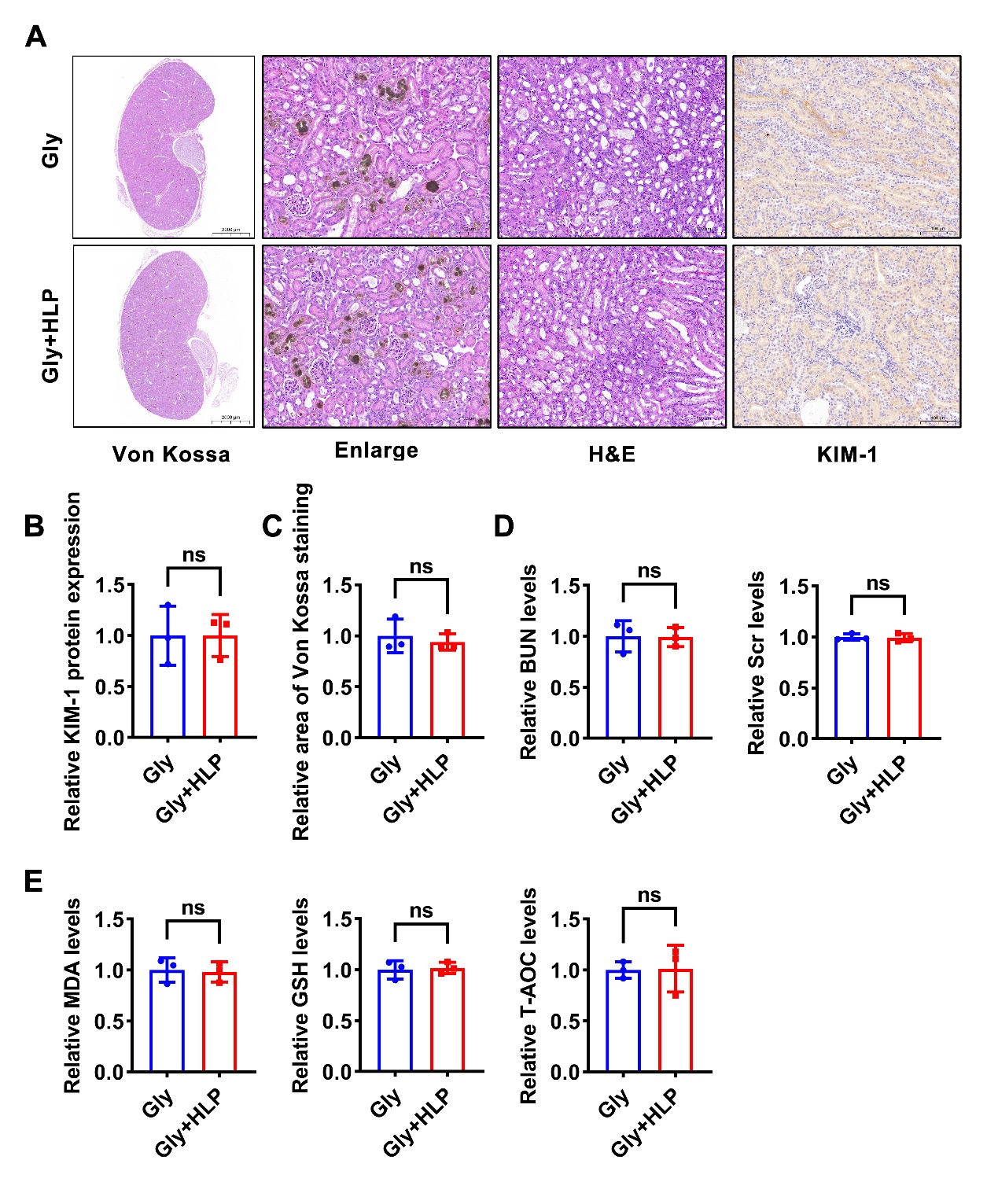
Figure S1 Free HLP alone does not ameliorate glyoxylate-induced kidney injury.**

(A) Representative images of Von Kossa staining (whole kidney and magnified view), H&E staining, and KIM-1 immunohistochemistry from the Gly and Gly+HLP groups. (B) Quantitative analysis of KIM-1 protein expression. (C) Quantitative analysis of Von Kossa positive crystal area. (D) Quantitative analysis of serum BUN and Scr levels. (E) Quantitative analysis of renal MDA, GSH, and T-AOC levels. ns: no statistical significance.


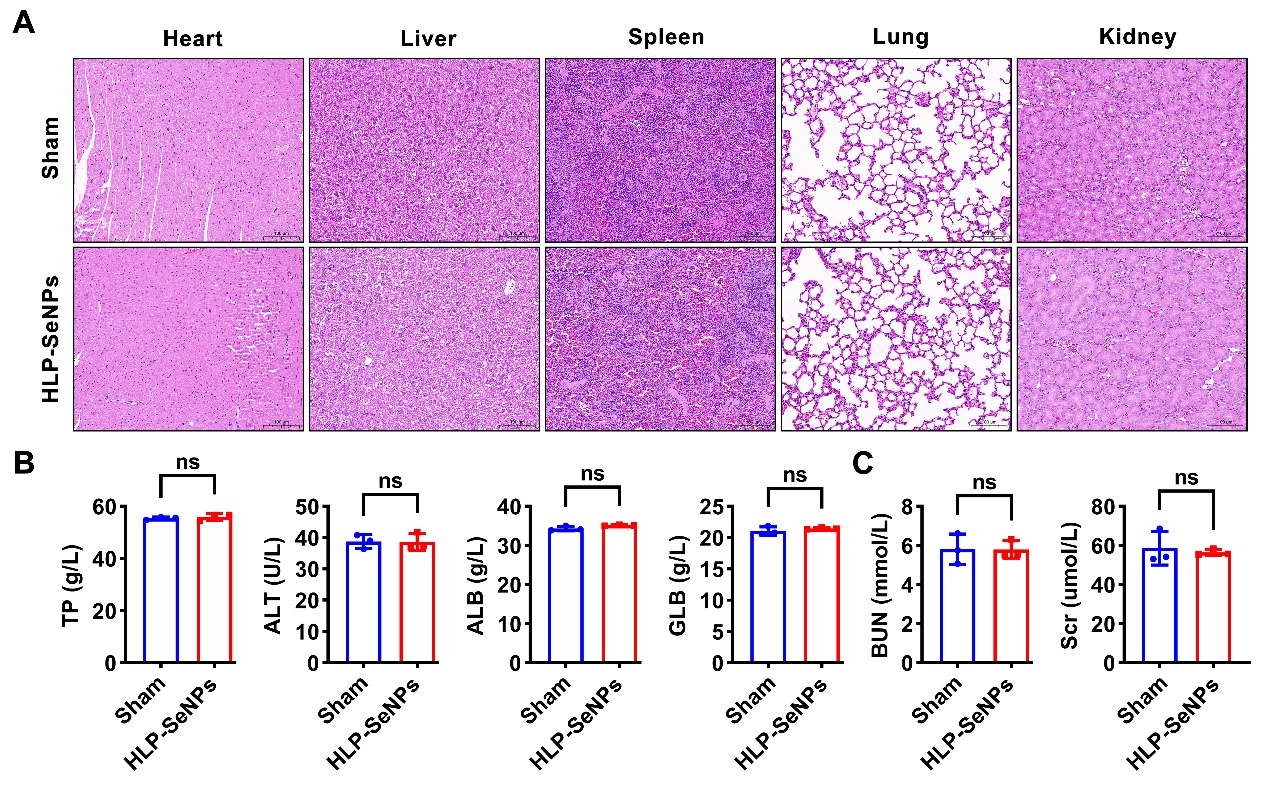
**Figure S2 Biosafety evaluation of HLP-SeNPs administration in healthy mice.**

(A) Representative H&E staining of major organs (heart, liver, spleen, lung, and kidney) from the Sham and HLP-SeNPs groups. (B-C) Serum biochemical indicators of organ function: (B) TP, ALT, ALB, GLB for hepatic function; (C) BUN and Scr for renal function. All comparisons showed no statistical significance (ns).

**
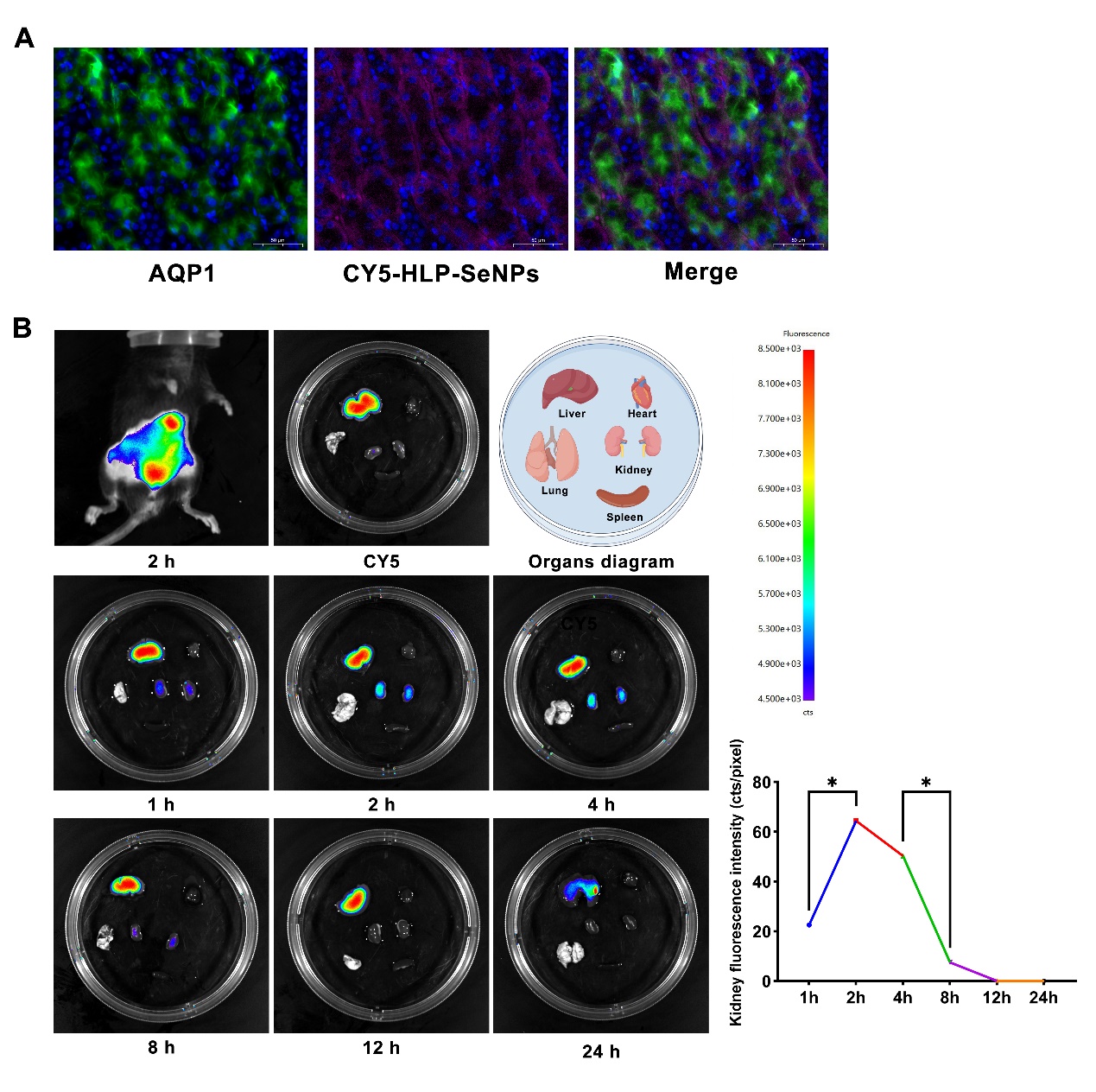
Figure S3 In vivo biodistribution and renal targeting of HLP-SeNPs after oral administration.**

(A) Immunofluorescence co-localization: Cy5-labeled HLP-SeNPs (purple) co-localizes with proximal tubular marker AQP1 (green); DAPI (blue) stains cell nuclei. (B) In vivo and ex vivo fluorescence imaging of Cy5-HLP-SeNPs at different time points post-administration, with a dissected organ schematic and quantitative analysis of renal fluorescence intensity over time. *p < 0.05.

**
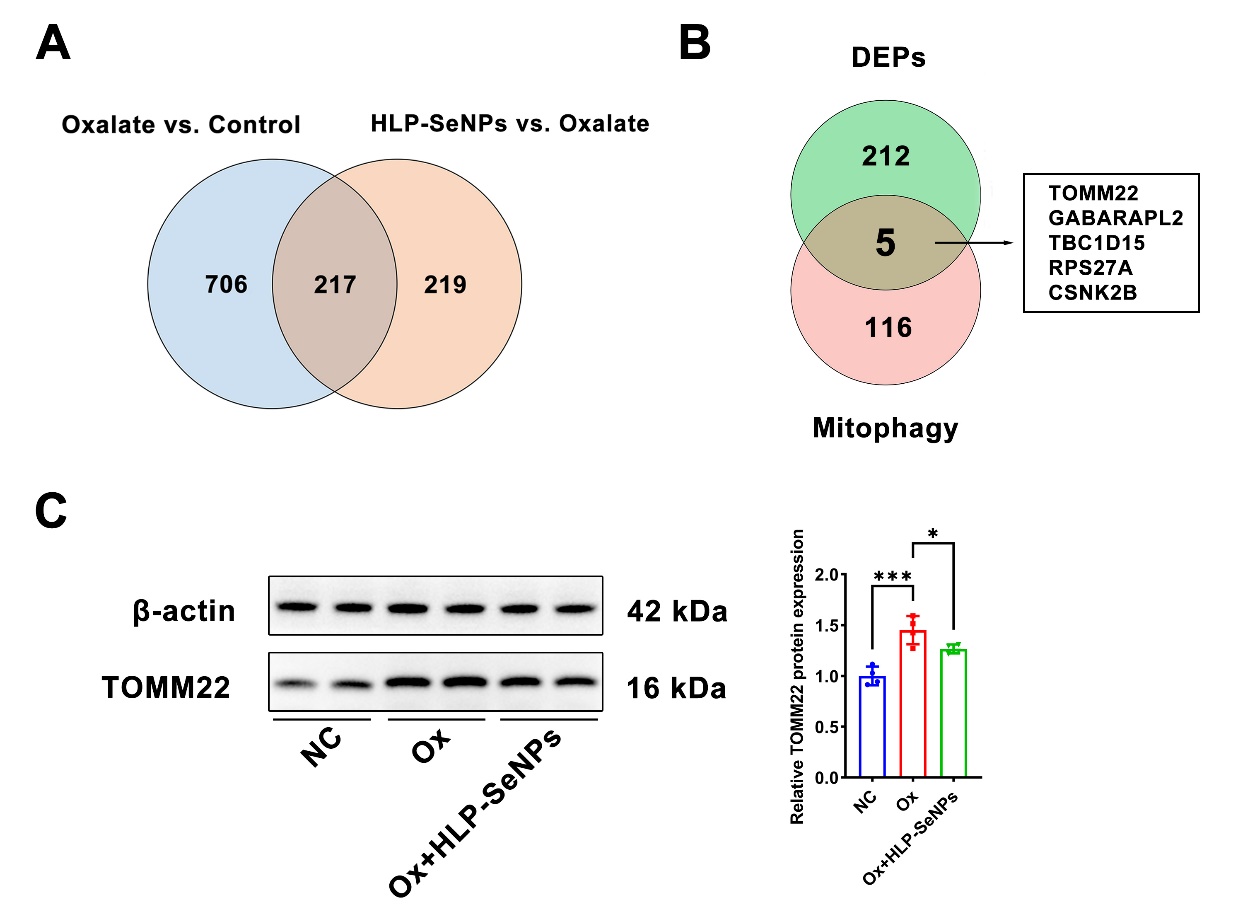
Figure S4 Intersection analysis screens mitophagy-related candidate targets**

(A) Venn diagram showing overlapping DEPs between Oxalate vs. Control and HLP-SeNPs vs. Oxalate comparisons. (B) Intersection of reversed DEPs with mitophagy-related gene sets from GSEA database, identifying 5 candidate target proteins. (C) Western blot result (left) and semi-quantitative analysis (right) of TOMM22 protein expression in HK-2 cells from different groups. **P < 0.001, *P < 0.05. NC: Normal control, Ox: Oxalate.

**
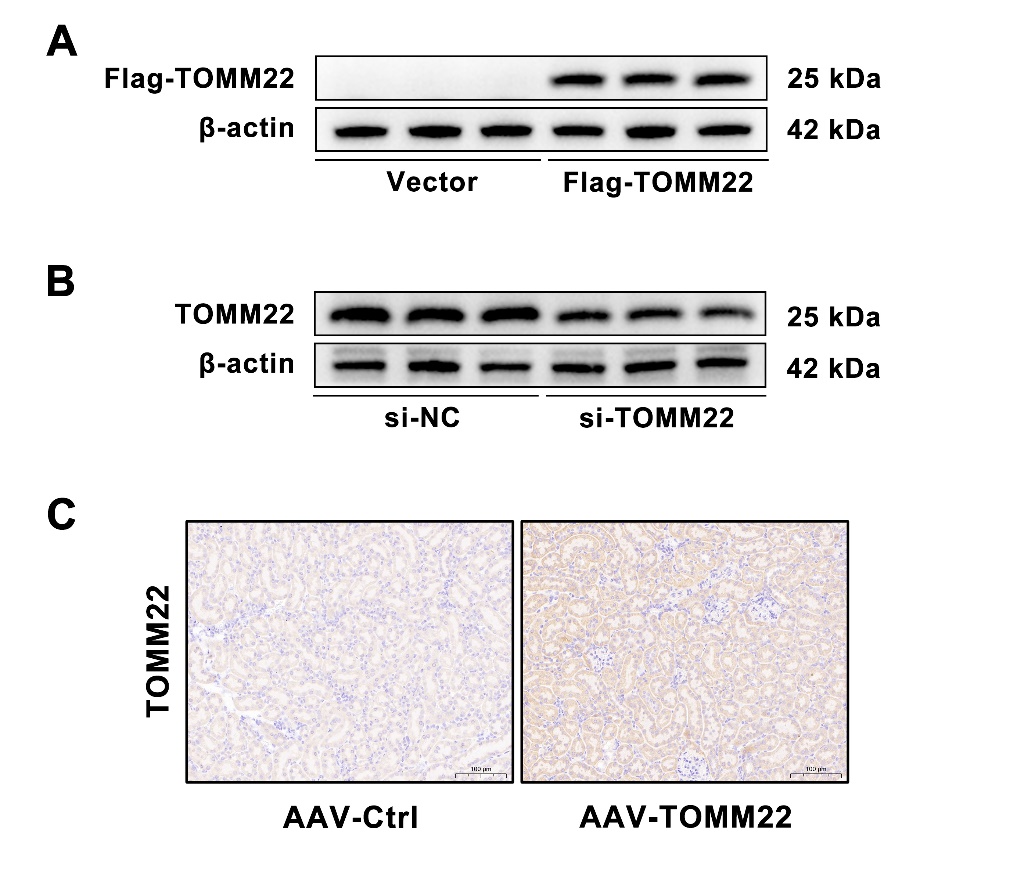
Figure S5 Validation of TOMM22 overexpression and knockdown efficiency**

(A) Western blot verification of Flag-TOMM22 overexpression efficiency in vitro; (B) Western blot verification of TOMM22 knockdown efficiency in cells transfected with si-TOMM22; (C) Immunohistochemical staining validation of TOMM22 overexpression efficiency in mouse kidney tissues after AAV-TOMM22 injection.
